# Quantum-assisted fragment-based automated structure generator (QFASG) for small molecule design: an *in vitro* study

**DOI:** 10.3389/fchem.2024.1382512

**Published:** 2024-04-03

**Authors:** Sergei Evteev, Yan Ivanenkov, Ivan Semenov, Maxim Malkov, Olga Mazaleva, Artem Bodunov, Dmitry Bezrukov, Denis Sidorenko, Victor Terentiev, Alex Malyshev, Bogdan Zagribelnyy, Anastasia Korzhenevskaya, Alex Aliper, Alex Zhavoronkov

**Affiliations:** ^1^ Insilico Medicine Hong Kong Ltd., Hong Kong, Hong Kong SAR, China; ^2^ Insilico Medicine AI Limited, Abu Dhabi, United Arab Emirates

**Keywords:** fragment-based drug design, kinase inhibitors, computer-assisted drug design, *de novo* design, molecular modeling, xTB, ATM, CAMKK2

## Abstract

**Introduction:** The significance of automated drug design using virtual generative models has steadily grown in recent years. While deep learning-driven solutions have received growing attention, only a few modern AI-assisted generative chemistry platforms have demonstrated the ability to produce valuable structures. At the same time, virtual fragment-based drug design, which was previously less popular due to the high computational costs, has become more attractive with the development of new chemoinformatic techniques and powerful computing technologies.

**Methods:** We developed Quantum-assisted Fragment-based Automated Structure Generator (QFASG), a fully automated algorithm designed to construct ligands for a target protein using a library of molecular fragments. QFASG was applied to generating new structures of CAMKK2 and ATM inhibitors.

**Results:** New low-micromolar inhibitors of CAMKK2 and ATM were designed using the algorithm.

**Discussion:** These findings highlight the algorithm’s potential in designing primary hits for further optimization and showcase the capabilities of QFASG as an effective tool in this field.

## 1 Introduction

Generative chemistry is an emerging trend in drug design that is gradually replacing virtual screening of existing compounds. Most state-of-the-art platforms for *de novo* drug design now employ deep learning (DL) solutions. While DL algorithms are a valuable technique, they often lack interpretability, making it challenging to understand the rationale behind their decisions. Recently published algorithms also suffer from a lack of medicinal chemistry expertise, resulting in the generation of chemically insufficient, unstable, toxic, inactive, or previously published structures (Ivanenkov et al., 2023b).

On the other hand, there is currently no *in vitro* approved fully automated algorithm available that can generate small-molecule structures in a medicinal chemistry-driven manner that considers factors such as ligand‒protein interactions and structural complexity. Virtual fragment-based drug design (VFBDD), an approach that has existed for several decades (Kumar et al., 2012), holds great potential but remains underestimated and underutilized in the pharmaceutical industry. One of the key advantages of VFBDD is its capacity to explore a vast chemical space using a relatively small number of fragments. By focusing on smaller molecular fragments, this approach enables more efficient screening and optimization of potential drug candidates. Recent examples of such algorithms include MoleGear (Chu and He, 2019), AutoGrow (Spiegel and Durrant, 2020), and GenUI (Sicho et al., 2021).

However, current methods in VFBDD have certain limitations that hinder their widespread adoption (see Supplementary Table S1). Many known methods have been developed for modifying prepositioned scaffolds within a binding site and are not suitable for *de novo* design. Other problems with VFBDD include low diversity of generated structures, producing structures that are unfavourable from the perspective of medicinal and organic chemistry, and high computational cost, especially when combined with large libraries of fragments, resulting in a combinatorial exposure problem and an inability to explore the requisite chemical space. Another challenge lies in hit selection due to difficulties in accurately estimating binding affinity, synthetic accessibility, drug-likeness, etc.

Many of these limitations can be overcome by employing modern chemoinformatic techniques. This article introduces QFASG, a new method for *de novo* structure generation that utilizes advanced chemoinformatic solutions and adheres to medicinal chemistry rules. The algorithm and its application in generating new inhibitors of ATM and CAMKK2 kinases are discussed.

## 2 QFASG pipeline

**FIGURE 1:**
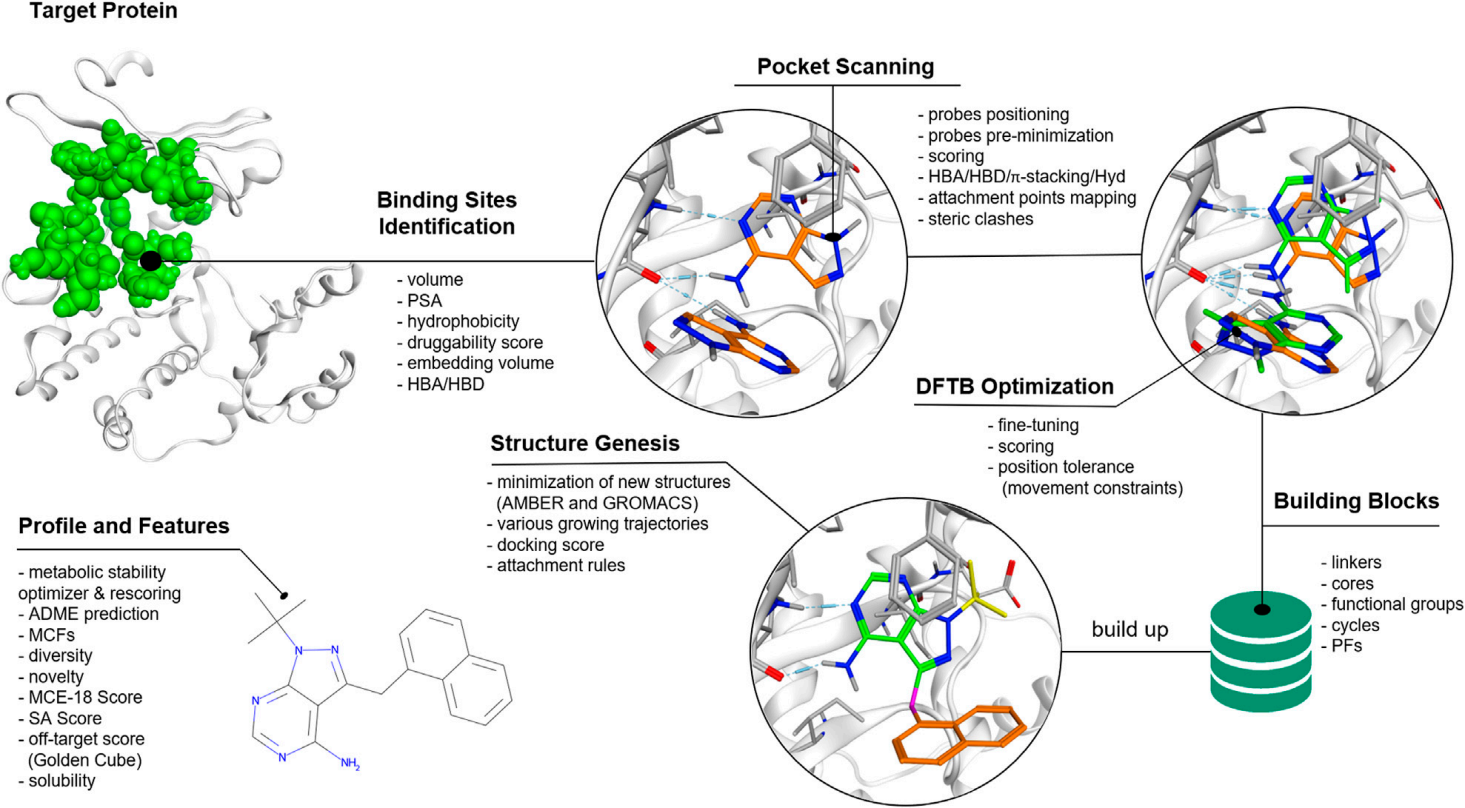
QFASG pipeline. The algorithm consists of several consequent steps, including binding site identification, probe positioning, structure generation, and analysis of generated structures.

The QFASG pipeline comprises several interconnected modules. Functionally, the algorithm can be divided into two parts: the non-iterative section focuses on placing probes (initial fragments) within the target binding site, while the second (iterative) part facilitates fragment-wise structural growth (Figure 1).

### 2.1 Probe placement

Probe placement is conducted through four steps: binding site selection, positioning of probes based on pharmacophore analysis, fine-tuning of probe positions using semiempirical extended tight-binding GFN2-xTB (Bannwarth et al., 2019), and final probe selection. The identification of the binding site is performed based on the position of the reference ligand or *de novo* based on characteristics such as pocket volume, polar surface area (PSA), hydrophobicity, etc. (Ivanenkov et al., 2023a). Probes are positioned within the binding sites, targeting protein hotspots such as hydrogen bond donors/acceptors and aromatic rings. The precise adjustment of probe positions is achieved using the tight-binding quantum chemical GFN2-xTB method. Due to short-range damped interactions of cumulative atomic multipole moments in this method, the description of non-covalent binding is achieved without the use of additional corrections. As it was shown (Bannwarth et al., 2019) the accuracy of this method is better or comparable to other semiempirical methods, however in our preliminary experiments GFN2-xTB turned out to be the fastest. It can be assumed that using more accurate quantum chemical methods in the pipeline, e.g., DFT with van der Waals corrections, would improve the quality of ligand prediction, but this is still beyond computational capabilities. Potentially, the quality of the structure generation model can be improved by using all the information obtained by the quantum chemical calculation since this information contains everything on which the ligand-pocket interaction depends. Though, such a stream of incoming data would greatly increase the cost of the model and an additional thorough analysis of the correlations in these data would be required. In the QFASG pipeline implementation of GFN2-xTB in xtb package (https://github.com/grimme-lab/xtb/releases) was used. After establishing probe placement, the best probes are selected based on pharmacophore scores and calculated binding energies.

### 2.2 Structural growth

The iterative part consists of several modules: linking, conformer generation, alignment, rigid docking, and selection. Initially, new structures are generated by growing fragments from the non-iterative part or previous iteration. These generated structures undergo evaluation using medicinal chemistry filters (MCFs) integrated into the Chemistry42 platform (Ivanenkov et al., 2023a). Subsequently, conformations of the new structures are generated using an algorithm available in Chemistry42 (Ivanenkov et al., 2023a). The generated conformers are then aligned to their initial positions using the RDKit (RDKit, 2022) function “GetBestRMS”. The obtained positions are further optimized using rigid docking techniques implemented in Chemistry42 (Ivanenkov et al., 2023a).

Finally, a selection process is performed on the generated compounds based on one of two scoring functions: calculated free binding energy obtained from rigid docking or PLI-score (protein‒ligand interaction score) (Ivanenkov et al., 2023a). Additionally, there are two options for how the selection can be performed: with or without interaction fingerprint (IFP) clustering. Selection without IFP clustering aims to identify structures with the highest score, but it may result in selecting structures with high structural similarity and similar binding modes.

Selection with IFP clustering involves grouping the generated structures based on their binding mode and ligand‒protein interaction network. From each cluster, the structure with the best score is selected. This approach allows users to obtain a diverse set of structures with various binding modes.

Once an iteration is completed, the selected structures can be used as starting points for the next iteration until generation stops due to limitations such as molecular weight or exceeding the maximum number of iterations.

To generate the output file, structures from all iterations undergo a rigorous evaluation using an enhanced version of the MCFs. These filters consider not only the core structure but also the terminal functional groups, in addition to other user-defined conditions such as molecular weight. Furthermore, various additional descriptors are computed, including ligand shape similarity, PLI score, and ReRSA score.

### 2.3 Linking rules

**FIGURE 2:**
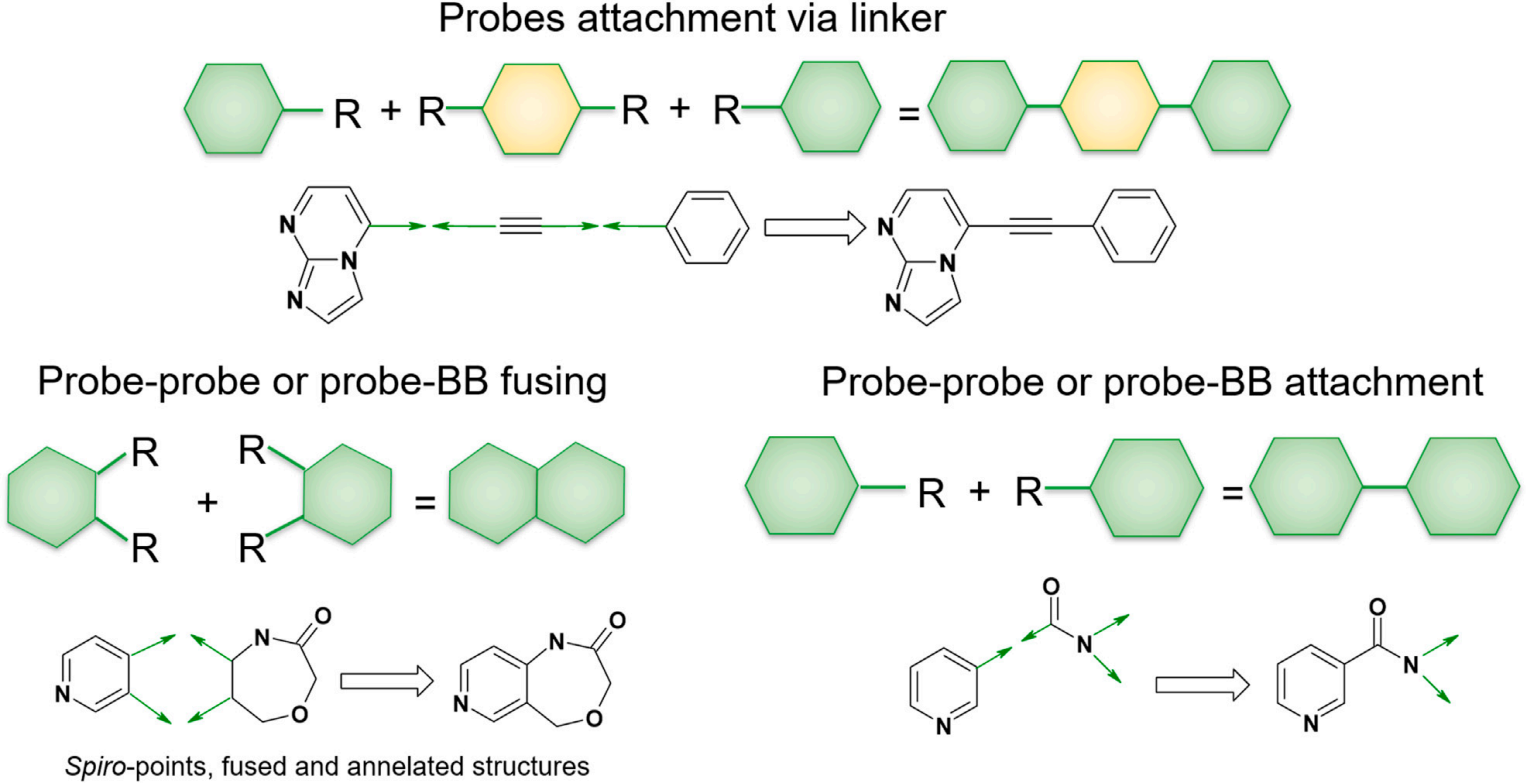
Linking rules. The QFASG algorithm allows several types of fragment linking, such as direct linking, linker-mediated attachment or ring fusing.

In QFASG, we employ several types of structure growing. First, we enable the direct linkage between two fragments through a single bond, which applies to both probe–probe and probe–fragment pairs. Second, the connection of two fragments can be achieved by employing a linking fragment. This rule is specifically designed for connecting two probes using a linker from the fragment library. Another possibility is the merging of rings from different fragments, resulting in the formation of condensed systems. Additionally, the formation of aliphatic ring linkages is allowed, leading to the generation of *spiro*-cycles. The last two rules are applicable to both probe–probe and probe–fragment pairs (Figure 2).

## 3 Potential of targeting CAMKK2 and ATM for cancer treatment

ATM (Ataxia Telangiectasia Mutated) and CAMKK2 (Calcium/Calmodulin-Dependent Protein Kinase Kinase 2) kinases play crucial roles in the pathophysiology of cancer and have emerged as promising targets for cancer treatment. ATM is a member of the phosphatidylinositol 3-kinase-related kinase family, which also includes ataxia- and rad3-related kinase (ATR), DNA-dependent protein kinase (DNA-PK), etc. (Blackford and Jackson, 2017). ATM kinase is involved in the DNA damage response and repair: it participates in protecting DNA from double-strand breaks and reactive oxygen species induced by radio- and chemotherapy, making ATM inhibition a promising approach to sensitize cancer cells (Mei et al., 2019). ATM can be considered as a target for treatment of solid tumours, glioblastoma, sarcoma, lung, prostate and colorectal cancer and other pathologies (McCabe et al., 2015; Shu et al., 2023). Currently, Merck, Pfizer, and AstraZeneca are conducting early-stage clinical trials of several ATM inhibitors (Table 1) for potential application as monotherapy or combined cancer treatment (Ahmed and Tuxworth, 2022; Waqar et al., 2022; Zimmermann et al., 2022).

**TABLE 1:** Summary data on ATM inhibitors in clinical trials.

ClinicalTrials.gov ID	Name	Structure	Phase	Indication	Company name
NCT05917145	WSD-0628	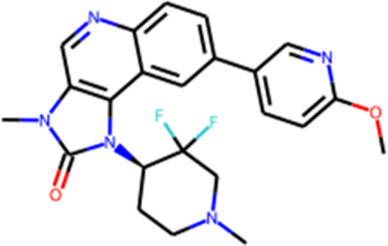	Phase I	Brain Cancer Therapy Glioblastoma Multiforme Therapy	WayShine Biopharm
NCT05002140	XRD-0394	Not disclosed	Phase I	Solid Tumor Therapy	XRad Therapeutics
NCT04882917	M-4076	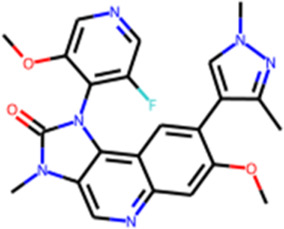	Phase I	Antineoplastic Enhancing Agents Solid Tumor Therapy	Merck KGaA
NCT03225105	M-3541	Not disclosed	Phase I	Antineoplastic Enhancing Agents Solid Tumor Therapy	Merck KGaA
NCT03423628	AZD-1390	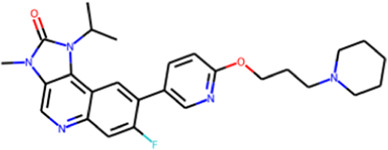	Phase I	Glioblastoma Multiforme Therapy Sarcoma Therapy	AstraZeneca
NCT05678010					
NCT02588105	AZD-0156	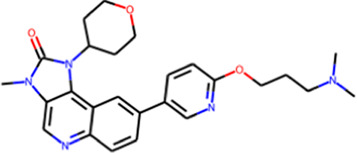	Discontinued	Solid Tumor Therapy	AstraZeneca

Calcium (Ca^2+^)/calmodulin-dependent protein kinase kinase 2 (CAMKK2) is a serine/threonine kinase that belongs to a family of Ca^2+^/CaM-dependent protein kinases. CAMKK2 regulates the activity of CAMK1, CAMK4, and AMP-activated protein kinase (AMPK) and plays a significant role in various physiological and pathological processes, including insulin signalling, metabolic homeostasis, inflammation, and cancer cell growth (Racioppi and Means, 2012). CAMKK2 is overexpressed in multiple tumour types such as prostate, breast, ovarian, gastric and hepatic cancers (O’Byrne et al., 2020), and its knockdown or inhibition leads to a decrease in cell proliferation and tumorigenicity, making CAMKK2 an attractive target for cancer treatment (Subbannayya et al., 2015). Several CAMKK2 inhibitors have been previously identified, including STO-609 and GSK650394 (Ma et al., 2016; Price et al., 2018).

## 4 Results and discussion

### 4.1 Reproducing binding modes of known protein ligands

**FIGURE 3:**
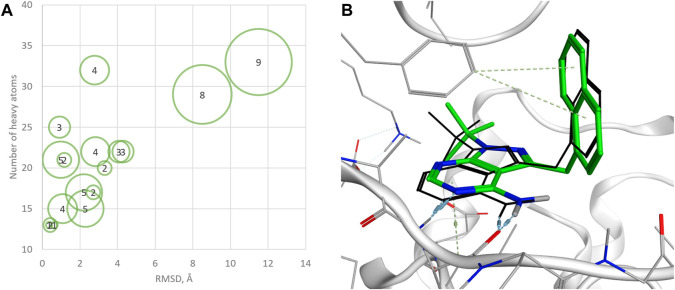
**(A)** Statistics on successful reproduction of the binding mode for known ligand‒protein complexes (sizes of green circles and numbers inside reflect the number of rotatable bonds); **(B)** example of reproduced structure (PDB ID—4GKI). Ligand crystal pose is shown in black sticks.

To evaluate the capability of QFASG in reproducing known protein ligand structures, we curated a dataset of 17 ligand‒protein complexes. The dataset was preprocessed using MOE v. 2022.02 [MOE (The Molecular Operating Environment), 2022], and the ligands were fragmented by breaking ring-connected bonds with RDKit (RDKit, 2022). Subsequently, we selected the fragments that formed hydrogen bonds with the protein as probes while collecting all obtained fragments as building blocks for structure generation. Our results showed that QFASG successfully reproduced crystal poses for 7 out of 17 complexes (with a root mean square deviation (RMSD) of less than 2 Å), almost reproduced poses for 8 complexes (with an RMSD between 2 Å and 4.5 Å), and failed to reproduce poses for 2 complexes (with an RMSD higher than 4.5 Å) (Figure 3, Supplementary Data S2, Supplementary Table S2).

Further analysis revealed that the accuracy of QFASG may be influenced by the number of heavy atoms in the ligand structures. Specifically, QFASG demonstrated a high success rate in reproducing binding modes for small molecules containing up to 25 heavy atoms. However, the results were less stable for structures composed of approximately 30 heavy atoms. A deeper examination of these examples highlighted that the accuracy of reproduction may also depend on the number of rotatable bonds (any single non-ring bond, attached to a non-terminal, non-hydrogen atom, and non-amide C-N bond), as finding an appropriate binding site for a flexible starting fragment presents a complex challenge. Perhaps the use of more sophisticated algorithms, such as diffusion models (Corso et al., 2022), will improve the quality of primary probe positioning in the future.

### 4.2 Generating structures of new kinase inhibitors

#### 4.2.1 CAMKK2

**FIGURE 4:**
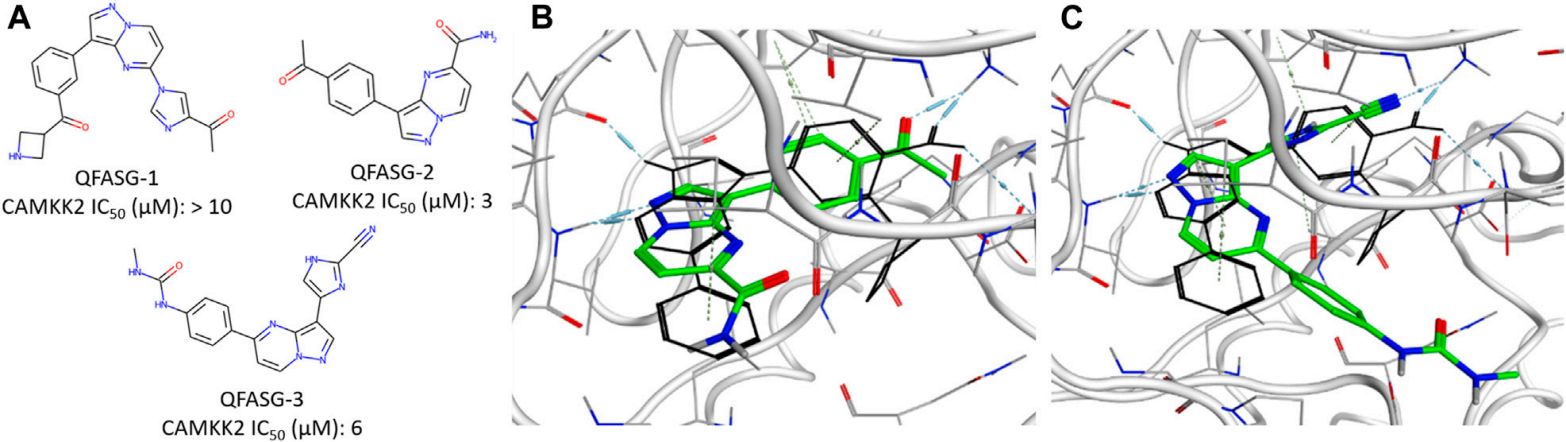
**(A)** Chemical structures generated by QFASG and their CAMKK2 inhibition activity **(B)** Predicted binding pose for QFASG-2 with CAMKK2 **(C)** Predicted binding pose for QFASG-3 with CAMKK2. Reference ligand is shown in black sticks.

To evaluate our model, we generated structures for new CAMKK2 inhibitors. The crystal structure of CAMKK2 in complex with GSK650394 (PDB ID - 6BKU) was used as a template. We selected the widely explored kinase hinge binder pyrazolo[1,5-a]pyrimidine (Bullock et al., 2005; Mathison et al., 2020) as a probe to serve as a starting point for structural growth. Our algorithm positioned this probe in two possible variants, both forming H-bond contacts with the crucial hinge region interaction. To assemble the fragment library, we selected 43 fragments commonly found in modern kinase inhibitors (Supplementary Figure S7). We performed four iterations of structural growth, selecting the best structures based on the calculated binding energy (Supplementary Data S3). Furthermore, we computed descriptors such as PLI score and shape similarity to a reference ligand for all the generated compounds. Consequently, a total of 465 structures were generated *de novo* (Supplementary Figure S8), and 10 structures were selected for expert analysis based on their PLI score, similarity to the reference ligand in shape, and formation of H-bonds with the backbone of Val^270^ and side chain of Lys^194^. Finally, three structures (QFASG-1, QFASG-2, and QFASG-3) were selected for synthesis and *in vitro* evaluation, taking into consideration a comprehensive assessment of the aforementioned descriptors, structural diversity, and expert evaluation regarding the presumed binding mode and medicinal chemistry perspectives. Novelty assessment did not reveal any structural analogs of QFASG-1 and QFASG-3. At the same time, QFASG-2 demonstrated partial substructure match with compounds previously reported as RET^V804M^ kinase inhibitors (Newton et al., 2020). As a result, all three compounds were successfully synthesized (Supplementary Data S1), and their CAMKK2 IC_50_ values were measured *in vitro* (Supplementary Figures S1–S3). Two out of the three compounds exhibited activity in the low micromolar range (3 μM with hill slope of −0.85 and 6 μM with hill slope of −1.05 for QFASG-2 and QFASG-3, respectively), while the IC_50_ value for QFASG-1 exceeded 10 μM (Figure 4). It should be noticed that compounds were tested with ATP concentration of 10 µM and thus could demonstrate lower activity when tested with ATP concentration equal to Km.

Regarding the results of the CAMKK2 *in vitro* study, there are several possibilities explaining the poor activity of one of the synthesized compounds. First, it is possible that the algorithm overestimates the activity of QFASG-1 due to the limited accuracy of the scoring functions. The other possibility is related to biochemical transformation, as QFASG-1 could undergo basic nitrogen oxidation or carbonyl group hydrolysis, producing a relatively stable geminal diol during storage, shipping, or testing. Finally, it should be noted that at this time, we performed a concept-approval (POC-study) test using small focused libraries of fragments and moderate settings (we did not calculate similarity to the reference compound in shape, and we used calculated free binding energy for intermediate selection). Additionally, our attention was mostly focused on selecting chemically simple structures with high synthetic feasibility. We expect that utilizing larger libraries of building blocks, using advanced settings (such as shape similarity and selecting intermediate structures using PLI-score), and selecting structures that are more chemically complex and that bind the targeted pocket in a more suitable manner could improve the performance of QFASG.

#### 4.2.2 ATM

**FIGURE 5:**
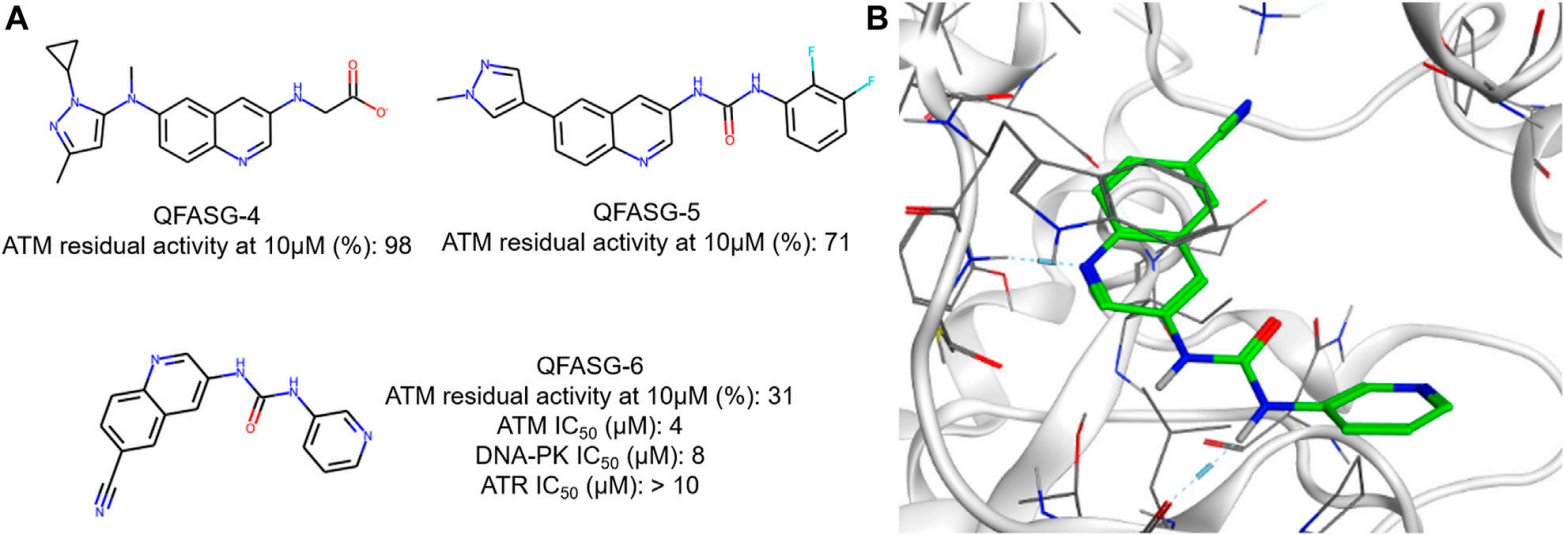
**(A)** Chemical structures generated by QFASG and their inhibitory activity against ATM, DNA-PK, and ATR kinases **(B)** Predicted binding pose for QFASG-6 with ATM.

Another round of generation was conducted with the aim of identifying the structures of new ATM inhibitors. In this case, the crystal structure of ATM in complex with the M4076 inhibitor (PDB ID—7NI4) was used as a template. We chose the 3-aminoquinoline core as an appropriate probe. The quinoline scaffold is well established in medicinal chemistry and kinase inhibitor design (Musiol, 2017; Zhou et al., 2020; Zaraei et al., 2022; Kim et al., 2023). Structurally diverse fragments (94 structures) were used as building blocks (Supplementary Figure S9). Through four iterations, 191 structures were generated (Supplementary Data S4, Supplementary Figure S10). Subsequently, three structures were selected for synthesis (Figure 5, Supplementary Data S1) and *in vitro* evaluation, following a similar approach as in the CAMKK2 case study. No structural analogs were detected for QFASG-4 and QFASG-5 during novelty assessment, while QFASG-6 was found in substructure of compounds reported as MALT1 inhibitors in 2020 (Pissot Soldermann et al., 2020). Among the three compounds tested, QFASG-6 exhibited an IC_50_ value of 4 μM with hill slope of −1.1. Furthermore, to assess the selectivity of this compound, we measured its activity against DNA-PK and ATR kinases. QFASG-6 demonstrated moderate activity against ATM and DNA-PK (IC_50_ value of 8 μM with hill slope of −0.85), while no ATR inhibition was observed (Supplementary Figures S4–S6). As well as for CAMKK2 assay, it should be noticed that ATP concentration was equal to 10 µM in this study.

The structures QFASG-5 and QFASG-6 exhibit considerable similarity, differing primarily in one functional group. Specifically, QFASG-5 features an N-methyl imidazole group, while QFASG-6 incorporates a nitrile group. Both functional groups have the potential to act as hydrogen bond acceptors and, presumptively, were designed by the algorithm to interact with a positively charged side chain of a Lys^2717^ residue. However, it is possible that the size of the N-methyl imidazole group within the QFASG-5 structure prevents optimal interaction with the lysine residue due to steric clashes.

## 5 Conclusion

In this paper, we presented QFASG, a fragment-based algorithm designed for the *de novo* generation of novel chemical structures. We demonstrated the platform’s ability to reproduce the reported crystal structures and to provide novel inhibitors with low-micromolar activity against pharmacologically relevant targets. QFASG offers a range of customizable settings, including probe and building block selection, filtering based on crucial interactions, clustering of generated structures using interaction fingerprints, and determination of the number of conformations to generate. These features allow for flexibility in experiment duration and results. Overall, we assess QFASG as a promising fragment-based approach that has been successfully validated *in vitro*. It holds potential for rational drug design, particularly in generating primary hit compound structures for target proteins or generating initial hypotheses for further exploration within AI-driven generative chemistry platforms.

## 6 Materials and methods

### 6.1 CAMKK2 kinase activity assay

The assay is available as a product of DiscoverX/Eurofins. CaMKK2 (h) was incubated with 20 mM Tris/HCl pH 8.5, 0.2 mM EDTA, 0.5% BSA, 0.5 mM CaCl2, 0.016 mg/mL calmodulin, 150 µM LSNLYHQGKFLQTFCGSPLYRRR, 10 mM MgAcetate and [gamma-33P]-ATP (specific activity and concentration of 10 µM). The reaction was initiated by the addition of the Mg/ATP mix. After incubation for 40 min 13 at room temperature, the reaction was stopped by the addition of phosphoric acid to a concentration of 0.5%. An aliquot of the reaction was then spotted onto a filter and washed four times for 4 min in 0.425% phosphoric acid and once in methanol prior to drying and scintillation counting.

### 6.2 ATM kinase activity assay

The assay is available as a product of DiscoverX/Eurofins. ATM (h) was incubated in assay buffer containing 30 nM GST-cMyc-p53 and Mg/ATP (concentration of 10 µM). The reaction is initiated by the addition of the Mg/ATP mix. After incubation for 30 min at room temperature, the reaction was stopped by the addition of stop solution containing EDTA. Finally, detection buffer was added, which contained a d2-labelled anti-GST monoclonal antibody and a europium-labelled anti-phospho Ser15 antibody against phosphorylated p53. The plate was then read in time-resolved fluorescence mode, and the homogeneous time-resolved fluorescence (HTRF) signal is determined according to the formula HTRF = 10000 x (Em665 nm/Em620 nm).

### 6.3 DNA-PK kinase activity assay

The assay is available as a product of DiscoverX/Eurofins. DNA-PK (h) was incubated in assay buffer containing 50 nM GST-cMyc-p53 and Mg/ATP (concentration of 10 µM). The reaction is initiated by the addition of the Mg/ATP mix. After incubation for 30 min at room temperature, the reaction was stopped by the addition of stop solution containing EDTA. Finally, detection buffer was added, which contained a d2-labelled anti-GST monoclonal antibody and a europium-labelled anti-phospho Ser15 antibody against phosphorylated p53. The plate is then read in time-resolved fluorescence mode, and the homogeneous time-resolved fluorescence (HTRF) signal is determined according to the formula HTRF = 10,000 × (Em665 nm/Em620 nm).

### 6.4 ATR kinase activity assay

The assay is available as a product of DiscoverX/Eurofins. ATR/ATRIP (h) was incubated in assay buffer containing 50 nM GST-cMyc-p53 and Mg/ATP (concentration of 10 µM). The reaction was initiated by the addition of the Mg/ATP mix. After incubation for 30 min at room temperature, the reaction was stopped by the addition of stop solution containing EDTA. Finally, detection buffer was added, which contained a d2-labelled anti-GST monoclonal antibody and a europium-labelled anti-phospho Ser15 antibody against phosphorylated p53. The plate is then read in time-resolved fluorescence mode, and the homogeneous time-resolved fluorescence (HTRF) signal was determined according to the formula HTRF = 10,000 × (Em665 nm/Em620 nm).

## Data Availability

The original contributions presented in the study are included in the article/Supplementary Material, further inquiries can be directed to the corresponding author.
