# Supplementary material for: Quantum-assisted fragment-based automated structure generator (QFASG) for small molecule design: an in vitro study
Source: Front Chem. 2024 Apr 3;12:1382512. doi: 10.3389/fchem.2024.1382512 (PMC11021760; doi:10.3389/fchem.2024.1382512)
Supplement: Supplementary file 1 [file DataSheet1.docx]

Supplementary Material

# Supplementary Data

**Supplementary Data 1**. Synthesis of compounds.

**QFASG-1**

**
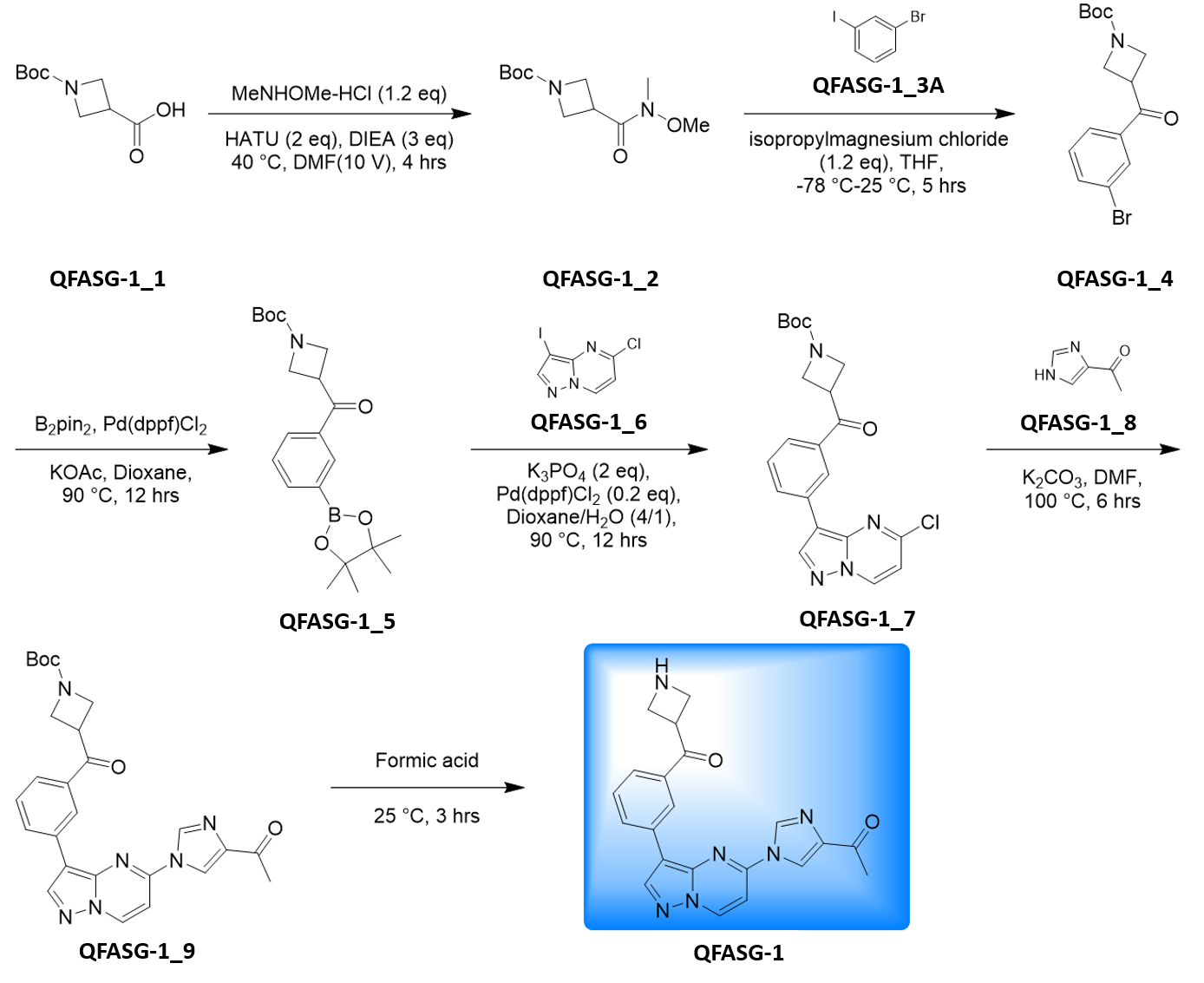
**

To a solution of QFASG-1_1 (10 g, 49.70 mmol, 1 eq) in N,N-dimethylformamide (60 mL) was added 2-(3H-[1,2,3]triazolo[4,5-b]pyridin-3-yl)-1,1,3,3-tetramethylisouronium hexafluorophosphate(V) (37.79 g, 99.39 mmol, 2 eq), N-ethyl-N-isopropylpropan-2-amine (19.27 g, 149.09 mmol, 25.97 mL, 3 eq) and N,O-dimethylhydroxylamine hydrochloride (3.04 g, 49.70 mmol, 1 eq) at 25 °C. The reaction mixture was heated to 40 °C and stirred at 40 °C for 4 hrs. TLC (petroleum ether/ethyl acetate = 4/1, R_f_ = 0.25) indicated starting material was consumed completely and desired spot was formed. One additional vial was set up as described above. After cooling to room temperature, all of two reaction mixtures were quenched by addition water (600 mL) at 0 °C and extracted with ethyl acetate (200 mL × 3). The combined organic layers were washed with brine (300 mL), dried over anhydrous sodium sulfate and filtered. The filtrate was concentrated under reduced pressure to give residue, which was purified by column on silica gel (eluted with petroleum ether/ethyl acetate = 70/30 to 65/35, R_f_ = 0.25) to get QFASG-1_2 (12 g, yield, 49.42%) as a yellow oil.

^1^H NMR: ET78201-5-P1N1, 400 MHz, CD_3_OD

δ = 4.11 - 4.01 (m, 4H), 3.85 - 3.74 (m, 1H), 3.70 (s, 3H), 3.20 (s, 3H), 1.44 (s, 9H)

To a solution of QFASG-1_3A (9.03 g, 31.92 mmol, 4.07 mL, 1.3 eq) in tetrahydrofuran (70 mL) was added isopropylmagnesium chloride (2 M, 14.73 mL, 1.2 eq) at -78 °C for 1 hr under nitrogen atmosphere and then added QFASG-1_2 (6.00 g, 24.55 mmol, 1 eq) at -78 °C – 25 °C. The reaction mixture was warmed to 25 °C and stirred at 25 °C for 5 hrs. TLC (petroleum ether/ethyl acetate = 3/1, R_f_ = 0.25) indicated starting material was consumed completely and desired spot was formed. The reaction mixture was quenched by addition aqueous ammonium chloride solution (100 mL) at 0 °C and extracted with ethyl acetate (40 mL × 3). The combined organic layers were washed with brine (50 mL), dried over anhydrous sodium sulfate and filtered. The filtrate was concentrated under reduced pressure to give residue, which was purified by column on silica gel (eluted with petroleum ether/ethyl acetate = 70/30 to 65/35, R_f_ = 0.25) to get QFASG-1_4 (7.5 g, yield, 89.79%) as a yellow oil.

^1^H NMR: ET78201-9-P1N1, 400 MHz, DMSO

δ = 8.00 (t, *J* = 1.7 Hz, 1H), 7.87 (dd, *J* = 1.8, 7.9 Hz, 2H), 7.51 (t, *J* = 7.9 Hz, 1H), 4.46 - 4.35 (m, 1H), 4.13 (br s, 2H), 3.96 (br t, *J* = 6.7 Hz, 2H), 1.37 (s, 9H)

To a solution of QFASG-1_4 (3.7 g, 10.88 mmol, 1 eq) in dioxane (80 mL) was added 4,4,4',4',5,5,5',5'-octamethyl-2,2'-bi(1,3,2-dioxaborolane) (4.14 g, 16.31 mmol, 1.5 eq), cyclopenta-2,4-dien-1-yl(diphenyl)phosphane;dichloropalladium;Fe^2+^ (1.59 g, 2.18 mmol, 0.2 eq) and potassium acetate (1.28 g, 13.05 mmol, 1.2 eq) at 25 °C. The reaction mixture was heated to 90 °C and stirred at 90 °C for 4 hrs under nitrogen atmosphere. ^1^H NMR showed starting material was consumed completely and the desired product was detected. After cooling to room temperature, the reaction mixture was quenched by addition aqueous ammonium chloride solution (100 mL) and extracted with ethyl acetate (40 mL × 3). The combined organic layers were washed with brine (50 mL), dried over anhydrous sodium sulfate and filtered. The filtrate was concentrated under reduced pressure to give residue, which was purified by column on silica gel (eluted with petroleum ether/ethyl acetate = 70/30 to 65/35, R_f_ = 0.25) to get QFASG-1_5 (4.2 g, yield 99.72%) as a yellow oil.

^1^H NMR: ET78201-13-P1N1, 400 MHz, DMSO

δ = 8.15 (s, 1H), 7.98 (td, *J* = 1.5, 7.9 Hz, 1H), 7.92 (d, *J* = 7.4 Hz, 1H), 7.56 (t, *J* = 7.6 Hz, 1H), 4.48 - 4.35 (m, 1H), 4.12 (br s, 2H), 3.96 (br t, *J* = 6.3 Hz, 2H), 1.37 (s, 9H), 1.31 (s, 12H)

To a solution of QFASG-1_5 (2 g, 5.16mmol, 1 eq) in dioxane (30 mL) and water (8 mL) was added QFASG-1_6 (1.73 g, 6.20 mmol, 1.2 eq) degassed and purged with nitrogen for 3 times, then added cyclopenta-2,4-dien-1-yl(diphenyl)phosphane;dichloropalladium;Fe^2+^ (755.74 mg, 1.03 mmol, 0.2 eq) and potassium phosphate (2.19 g, 10.33 mmol, 2 eq) at 25 °C. The reaction mixture was heated to 90 °C and stirred at 90 °C for 4 hrs under nitrogen atmosphere. The reaction mixture was heated to 90 °C and stirred at 90 °C for 12 hrs under nitrogen atmosphere. LCMS showed starting material was consumed completely and one major peak with desired mass was detected. After cooling to room temperature, the reaction mixture was quenched by addition aqueous ammonium chloride solution (100 mL) and extracted with ethyl acetate (40 mL × 3). The combined organic layers were washed with brine (50 mL), dried over anhydrous sodium sulfate and filtered. The filtrate was concentrated under reduced pressure to give residue, which was purified by column on silica gel (eluted with petroleum ether/ethyl acetate = 62/38 to 57/43, R_f_ = 0.25) to get QFASG-1_7 (800 mg, yield 37.52%) as a yellow oil.

^1^H NMR: ET78201-16-P1N1, 400 MHz, DMSO

δ = 9.24 (d, *J* = 7.3 Hz, 1H), 8.93 (s, 1H), 8.54 (t, *J* = 1.4 Hz, 1H), 8.32 - 8.28 (m, 1H), 7.79 - 7.75 (m, 1H), 7.67 - 7.59 (m, 1H), 7.24 (d, *J* = 7.3 Hz, 1H), 4.51 - 4.40 (m, 1H), 4.22 (br d, *J* = 1.1 Hz, 2H), 4.09 - 4.01 (m, 2H), 1.38 (s, 9H)

To a solution of QFASG-1_7 (400 mg, 968.83 μmol, 1 eq) in N,N-dimethylformamide (6 mL) was added potassium carbonate (267.80 mg, 1.94 mmol, 2 eq) at 25 °C. The reaction mixture was heated to 100 °C and stirred at 100 °C for 6 hrs. LCMS showed starting material was consumed completely and one major peak with desired mass was detected. One additional vial was set up as described above. After cooling to room temperature, all of two reaction mixtures were quenched by addition water (40 mL) at 0 °C and extracted with ethyl acetate (10 mL × 3). The combined organic layers were washed with brine (15 mL), dried over anhydrous sodium sulfate and filtered. The filtrate was concentrated under reduced pressure to give residue, which was purified by column on silica gel (eluted with petroleum ether/ethyl acetate = 3/97 to 1/99, R_f_ = 0.25) to get QFASG-1_9 (300 mg, yield 31.82%) as a yellow oil.

^1^H NMR: ET78201-18-P1N1, 400 MHz, DMSO

δ = 9.46 (d, *J* = 7.5 Hz, 1H), 8.94 (s, 1H), 8.87 (d, *J* = 10.5 Hz, 2H), 8.67 (s, 1H), 8.42 (d, *J* = 7.8 Hz, 1H), 7.76 (br d, *J* = 7.5 Hz, 2H), 7.68 - 7.62 (m, 1H), 4.58 - 4.47 (m, 1H), 4.20 (br s, 2H), 4.03 (br d, *J* = 6.8 Hz, 2H), 2.53 (s, 3H), 1.38 (s, 9H)

A solution of QFASG-1_9 (300 mg, 616.62 μmol, 1 eq) in formic acid (12 mL) was stirred at 25 °C for 3 hrs. LCMS showed starting material was consumed completely and one major peak with desired mass was detected. The reaction mixtures were concentrated under reduced pressure to give a residue, which was purified by prep-HPLC to get QFASG-1 (50 mg, yield 20.98%) as a yellow solid.

Method of prep-HPLC:

Column: Phenomenex Luna C^18^ 100*30mm*3μm;

Mobile phase: [H_2_O (0.2% formic acid) - acetonitrile];

Gradient: 1%-35% B over 8.0 min.

^1^H NMR: ET78201-20-P1N11, 400 MHz, CD_3_CN

δ = 9.04 (d, *J* = 7.6 Hz, 1H), 8.69 (s, 1H), 8.67 - 8.65 (m, 2H), 8.62 (d, *J* = 1.3 Hz, 1H), 8.36 (d, *J* = 8.0 Hz, 1H), 8.34 (s, 1H), 7.76 (d, *J* = 7.8 Hz, 1H), 7.68 - 7.63 (m, 1H), 7.39 (d, *J* = 7.5 Hz, 1H), 4.68 (quin, *J* = 8.5 Hz, 1H), 4.32 (s, 2H), 4.30 (s, 2H), 2.56 (s, 3H)

**QFASG-2**

**
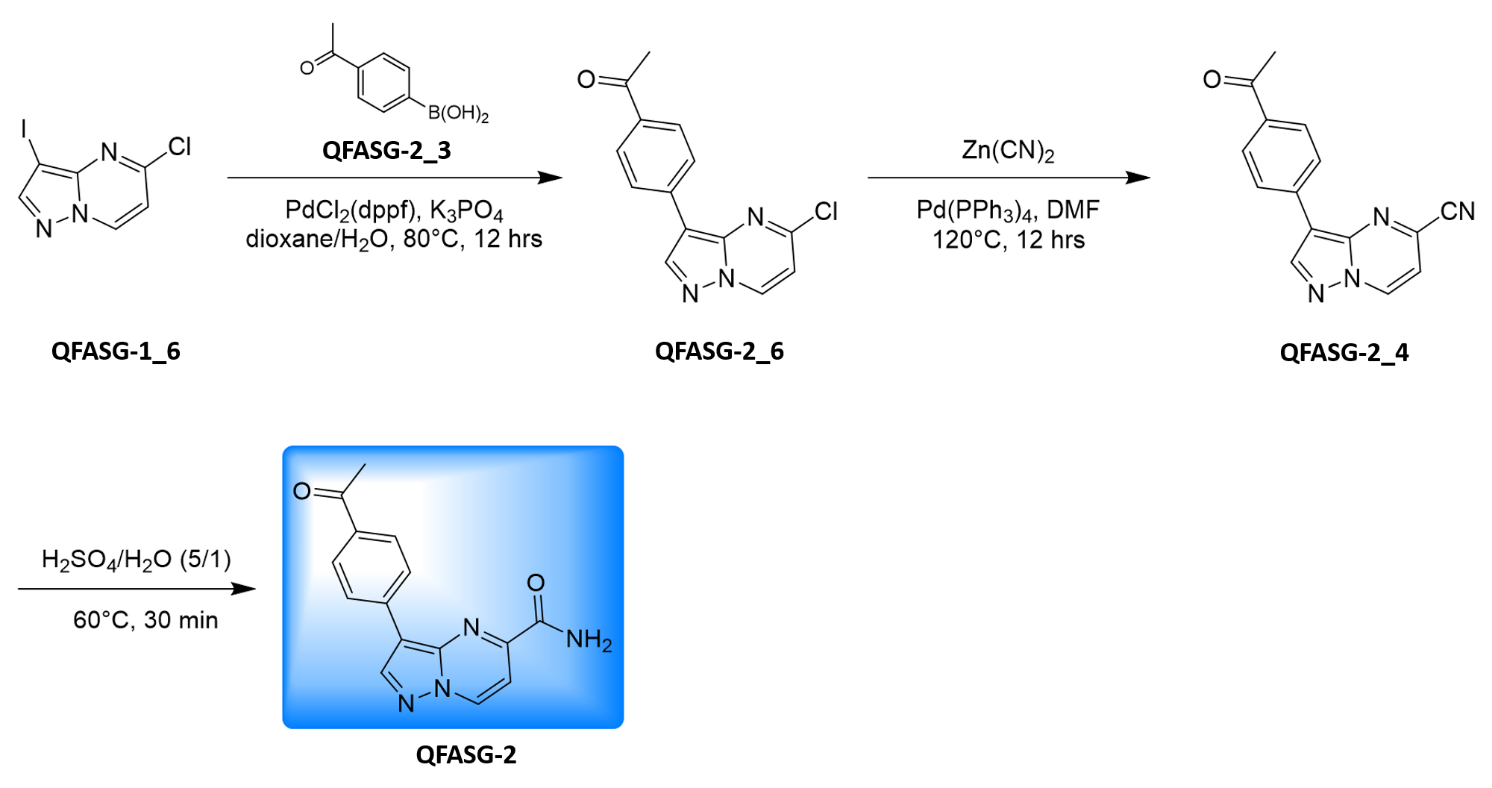
**

To a solution of QFASG-1_6 (2.5 g, 8.95 mmol, 1 eq) and QFASG-2_3 (1.47 g, 8.95 mmol, 1 eq) in a mixture of solvents dioxane (50 mL) and water (12.5 mL) were added [1,1-bis(diphenylphosphino)ferrocene]dichloropalladium(II) (1.31g, 1.79 mmol, 0.2 eq) and potassium phosphate (3.80 g, 17.89 mmol, 2 eq) at 25 °C under nitrogen. The reaction mixture warmed to 80 °C and stirred at 80 °C for 12 hrs. TLC (petroleum ether/ethyl acetate = 2/1, R_f_ = 0.31) showed starting material was consumed and desired spot was formed. One additional vial was set up as described above. After cooling to room temperature. All of two reaction mixtures were combined, diluted with water (100 mL × 2) and extracted with ethyl acetate (30 mL × 3). The combined organic layers were washed with brine (100 mL), dried over sodium sulfate, filtered and the filtrate was concentrated under reduced pressure to give a residue, which was purified by column on silica gel (eluted with petroleum ether/ethyl acetate = 100/0 to 70/30) to get QFASG-2_6 (2 g, yield 41.14%) as yellow solid.

^1^H NMR: ET78265-24-p1n1 400 MHz, CDCl_3_

δ = 8.60 (d, *J* = 7.3 Hz, 1H), 8.51 (s, 1H), 8.15 -8.09 (m, 2H), 8.07 - 8.02 (m, 2H), 6.89 (d, *J* = 7.3 Hz, 1H), 2.64 (s, 3H)

To a solution of QFASG-2_6 (500 mg, 1.84 mmol, 1 eq) in N,N-dimethylformamide (10 mL) was added zinc cyanide (432.18 mg, 3.68 mmol, 233.61 μL, 2 eq) and tetrakis(triphenylphosphine)palladium(0) (425.31 mg, 368.05 μmol, 0.2 eq) at 25 °C under nitrogen. The reaction mixture was heated to 120 °C and stirred at 120 °C for 12 hrs. TLC (petroleum ether/ethyl acetate = 1/2, R_f_ = 0.35) showed starting material was consumed and desired spot was formed. After cooling to room temperature, the reaction mixture was diluted with water (50 mL) and extracted with ethyl acetate (20 mL × 3). The combined organic phase was washed with brine (100 mL) and dried over sodium sulfate, filtered and the filtrate was concentrated under reduced pressure to get a crude. The crude was purified by column on silica gel (eluted with petroleum ether/ethyl acetate = 100 to 50/50) to get QFASG-2_4 (220 mg, yield 45.58%) as a yellow solid.

^1^H NMR: ET78265-25-p1n1 400 MHz, CDCl_3_

δ = 8.87 (d, *J* = 7.3 Hz, 1H), 8.69 (s, 1H), 8.21 - 8.15 (m, 2H), 8.13 - 8.07 (m, 2H), 7.18 (d, *J* = 7.1 Hz, 1H), 2.66 (s, 3H)

A solution of QFASG-2_4 (100 mg, 381.29 μmol, 1 eq) in a mixture of solvents sulfuric acid (0.5 mL) and water (0.1 mL) was heated to 60 °C and stirred at 60 °C for 30 min. LCMS showed starting material was consumed and desired Ms was detected. One additional vial was set up as described above. After cooling to room temperature. All of two reaction mixtures were combined and diluted with methanol (3 mL) and the suspension was filtered. The filtrate cake was dried over under reduced pressure to get a crude, which was purified by prep-HPLC to get QFASG-2 (21 mg, yield 9.83%) as a yellow solid.

Method of prep-HPLC:

Column: Phenomenex luna C^18^ 100*40mm*3μm;

Mobile phase: [water (0.2% formic acid) - acetonitrile];

Gradient: 20%-55% B over 8.0 min

^1^H NMR: ET78265-27-m1n1 400 MHz, DMSO

δ = 9.32 (d, *J* = 7.1 Hz, 1H), 9.03 (s, 1H), 8.45 (d, *J* = 8.4 Hz, 2H), 8.40 (br s, 1H), 8.04 (d, *J* = 8.4 Hz, 3H), 7.63 (d, *J* = 7.3 Hz, 1H), 2.61 (s, 4H)

**QFASG-3**

**
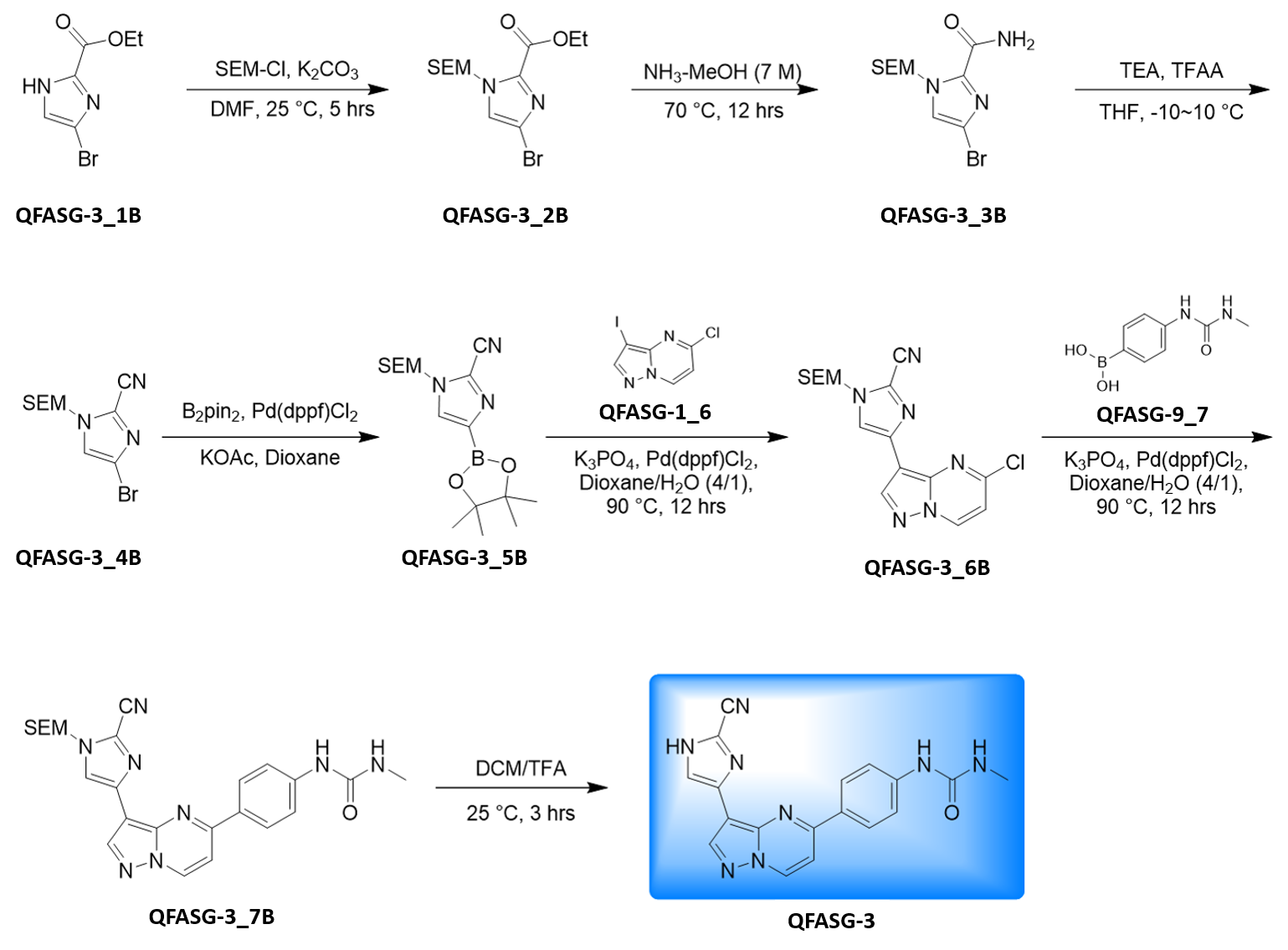
**

To the solution of QFASG-3_1B (13.4 g, 61.18 mmol, 1 eq) and potassium carbonate (10.15 g, 73.41 mmol, 1.2 eq) in dimethyl formamide (270 mL) was added 2-(trimethylsilyl)ethoxymethyl chloride (12.24 g, 73.41 mmol, 12.99 mL, 1.2 eq) at 0 °C. The reaction mixture was warmed to 25 °C and stirred at 25 °C for 5 hrs. LCMS showed starting material was consumed, 60% of desired compound was detected. The reaction mixture was diluted with water (600 mL) and extracted with ethyl acetate (1 L × 3). The combined organic layers were washed with brine (1 L) and dried over sodium sulfate, filtered and the filtrate was concentrated under reduced pressure to give a residue. The residue was purified by column on silica gel (eluted with petroleum ether/ethyl acetate=9/1, R_f_ = 0.70) to get QFASG-3_2B (9.2 g, yield 43.05%) as a yellow oil.

^1^H NMR: ET78141-33-P1N1, 400 MHz, CDCl_3_

δ = 7.25 (s, 1H), 5.76 (s, 2H), 4.41 (q, *J* = 7.1 Hz, 2H), 3.60 - 3.54 (m, 2H), 1.41 (t, *J* = 7.1 Hz, 3H), 0.96 - 0.90 (m, 2H), 0.00 - -0.04 (m, 9H)

A mixture of QFASG-3_2B (2.3 g, 6.58 mmol, 1 eq) in NH_3_/MeOH (7 M, 20 mL) was heated to 70 °C and stirred at 70 °C for 12 hrs. LCMS showed starting material was consumed, 90% of desired compound was detected. Three additional vials were set up as described above. After cooling to room temperature, all of four reaction mixture was filtered and the filtrate was concentrated under reduced pressure to give QFASG-3_3B (9 g, crude) as a yellow solid.

^1^H NMR: ET78141-35-P1N1, 400 MHz, CDCl_3_

δ = 7.23 (s, 1H), 5.86 (s, 2H), 3.64 - 3.50 (m, 2H), 1.00 - 0.91 (m, 2H), 0.06 - -0.06 (m, 9H)

To a solution of QFASG-3_3B (4 g, 12.49 mmol, 1 eq) in tetrahydrofuran (40 mL) was added triethylamine (3.16 g, 31.22 mmol, 4.35 mL, 2.5 eq) and trifluoroacetic anhydride (3.15 g, 14.99 mmol, 2.08 mL, 1.2 eq) at - 60 °C. Then the reaction mixture was warmed to 5 °C and stirred at 5 °C for 2 hrs. LCMS showed starting material was consumed, 90% of desired compound was detected. After warming to room temperature, the reaction mixture was diluted with water (120 mL) and extracted with ethyl acetate (150 mL × 3). The combined organic layers were washed with brine (300 mL) and dried over sodium sulfate, filtered and the filtrate was concentrated under reduced pressure to give a QFASG-3_4B (3.2 g, crude) as a brown oil.

^1^H NMR: ET78141-38-P1N1, 400 MHz, CDCl_3_

δ = 7.24 (s, 1H), 5.43 (s, 2H), 3.71 - 3.46 (m, 2H), 1.09 - 0.82 (m, 2H), 0.02 (s, 9H)

A mixture of QFASG-3_4B (2 g, 6.62 mmol, 1 eq) and 4,4,5,5-tetramethyl-2-(4,4,5,5-tetramethyl-1,3,2-dioxaborolan-2-yl)-1,3,2-dioxaborolane (2.52 g, 9.93 mmol, 1.5 eq) in dioxane (20 mL) was degassed and purged with nitrogen for 3 times, then the reaction mixture was added potassium acetate (1.30 g, 13.23 mmol, 2 eq) and cyclopentyl(diphenyl)phosphane;dichloropalladium;iron (968.37 mg, 1.32 mmol, 0.2 eq). The reaction mixture was heated to 90 °C and stirred at 90 °C for 12 hrs under N_2_ atmosphere. LCMS showed starting material was consumed, 47% of desired compound was detected. After cooling to room temperature, the reaction mixture was filtered to give the solution of QFASG-3_5B (2.1 g, crude) in dioxane (20 mL).

LCMS (ESI+): R_t_ = 0.449 min, m/z 268.2-boric acid (M+H)+.

A mixture of QFASG-1_6 (1.52 g, 5.44 mmol, 1 eq) and QFASG-3_5B (1.9 g, 5.44 mmol, 1 eq) in water (20 mL) and dioxane (80 mL) was degassed and purged with nitrogen for 3 times, then added cyclopentyl(diphenyl)phosphane;dichloropalladium;iron (796.00 mg, 1.09 mmol, 0.2 eq) and potassium phosphate (2.31 g, 10.88 mmol, 2 eq). The reaction mixture was heated to 90 °C and stirred at 90 °C for 12 hrs under nitrogen atmosphere. LCMS showed starting material was consumed, 20% of desired compound was detected. After cooling to room temperature, the reaction mixture was diluted with water (200 mL) and extracted with ethyl acetate (300 mL × 3). The combined organic layers were washed with brine (300 mL) and dried over sodium sulfate, filtered and the filtrate was concentrated under reduced pressure to give a residue. The residue was purified by column on silica gel (eluted with petroleum ether/ethyl acetate=0/1, R_f_ =0.20) to get QFASG-3_6B (400 mg, yield 19.62%) as a yellow solid.

^1^H NMR: ET78141-44-P1N1, 400 MHz, CD_3_OD

δ = 8.90 (d, *J* = 7.3 Hz, 1H), 8.55 (s, 1H), 8.04 (s, 1H), 7.06 (d, *J* = 7.3 Hz, 1H), 5.61 (s, 2H), 3.72 - 3.67 (m, 2H), 0.98 (t, *J* = 7.9 Hz, 2H), 0.03 - 0.00 (m, 9H)

A mixture of QFASG-3_7 (206.98 mg, 1.07 mmol, 1 eq) and QFASG-3_6B (400 mg, 1.07 mmol, 1 eq) in water (0.8 mL) and dioxane (3.2 mL) was degassed and purged with nitrogen for 3 times, then added cyclopentyl(diphenyl)phosphane;dichloropalladium;iron (156.14 mg, 213.39 μmol, 0.2 eq) and potassium phosphate (452.96 mg, 2.13 mmol, 2 eq). The reaction mixture was heated to 90 °C and stirred at 90 °C for 12 hrs under nitrogen atmosphere. LCMS showed starting material was consumed, 50% of desired compound was detected. After cooling to room temperature, the reaction mixture was diluted with water (5 mL) and extracted with ethyl acetate (10 mL × 3). The combined organic layers were washed with brine (20 mL) and dried over sodium sulfate, filtered and the filtrate was concentrated under reduced pressure to give a residue. The residue was purified by column on silica gel (eluted with ethyl acetate, R_f_ =0.20) to get QFASG-3_7B (290 mg, yield 55.63%) as a yellow solid.

^1^H NMR: ET78141-47-P1N1, 400 MHz, DMSO

δ = 9.16 (d, *J* = 7.5 Hz, 1H), 8.93 (s, 1H), 8.55 (s, 1H), 8.33 - 8.27 (m, 3H), 7.66 (dd, *J* = 8.2, 14.3 Hz, 3H), 6.18 (q, J = 4.4 Hz, 1H), 5.72 (s, 2H), 3.68 (t, *J* = 8.1 Hz, 2H), 2.70 (d, *J* = 4.5 Hz, 3H), 0.95 (t, *J* = 8.0 Hz, 2H), 0.06 - -0.06 (m, 9H)

To a solution of QFASG-3_7B (135 mg, 276.29 μmol, 1 eq) in dichloromethane (15 mL) was added trifluoroacetic acid (4.14 g, 36.35 mmol, 2.70 mL, 131.56 eq) at 0 °C. The reaction mixture was warmed to 25 °C and stirred at 25 °C for 3 hrs. LCMS showed 30% of desired Ms was detected. One additional vial was set up as described above. All of two reaction mixtures were concentrated under reduced pressure to give a residue, which was purified by prep-HPLC to get QFASG-3 (16 mg, yield 8.08%) as a yellow solid.

Method of prep-HPLC:

Column: Phenomenex Luna C^18^ 100*30mm*3μm;

Mobile phase: [H_2_O (0.2% formic acid)-acetonitrile];

Gradient: 10%-50% B over 8.0 min

^1^H NMR: ET78141-51-P1N3, 400 MHz, DMSO

δ = 9.00 (d, *J* = 7.5 Hz, 1H), 8.50 (s, 1H), 8.21 (d, *J* = 8.8 Hz, 2H), 8.03 - 7.87 (m, 1H), 7.62 (d, *J* = 7.5 Hz, 1H), 7.58 (d, *J* = 8.8 Hz, 2H), 2.65 (s, 3H)

**QFASG-4**


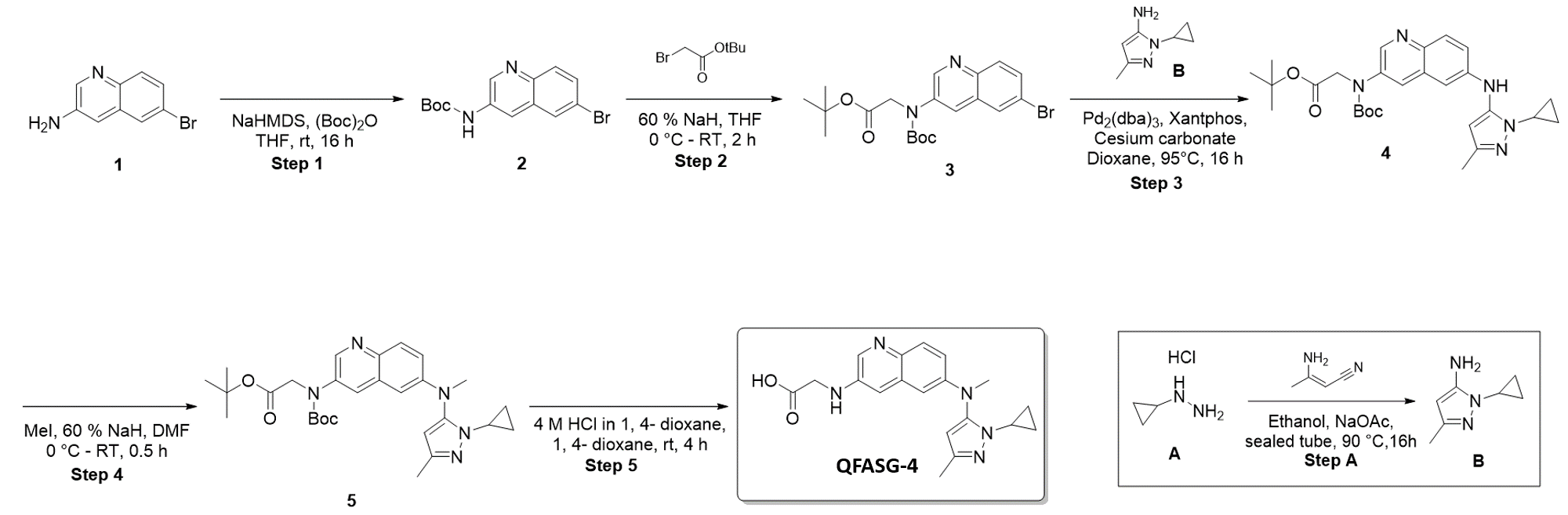


**Step-A**: Synthesis of 1-cyclopropyl-3-methyl-*1H*-pyrazol-5-amine (B):

To a stirred solution of cyclopropyl hydrazine hydrochloride (A) (500 mg, 4.629 mmol, 1.0 eq.) in ethanol (10 ml) was added (Z)-3-aminobut-2-enenitrile (379 mg, 4.629 mmol, 1.0 eq.) and sodium acetate (949 mg, 11.57 mmol, 2.5 eq.) in a sealed tube. The reaction mixture was stirred at 90° for 16h, after completion of the reaction (monitored by TLC) the reaction mass was concentrated on a rotavapor under reduced pressure. Obtained crude residue was diluted with EtOAc (70 mL) and washed with water (50 mL) and brine solution (50 mL). The organic layer was separated, dried over Na_2_SO_4_ and concentrated to obtained 1-cyclopropyl-3-methyl-*1H*-pyrazol-5-amine (B) as colorless liquid (300 mg crude, 76.35% pure).

LCMS (m/z): 76.35 % (M^+1^ = 138.06) at 2.80 RT.

**Step-1**: Synthesis of *tert*-butyl(6-bromoquinolin-3-yl) carbamate (2):

To a stirred solution of 6-bromoquinolin-3-amine (1) (500 mg, 2.24 mmol, 1.0 eq.) in THF (15 ml) was added 1M NaHMDS in THF (5.6 mL, 5.60 mmol, 2.5 eq.) followed by (Boc)_2_O (0.77 mL, 3.36 mmol, 1.5 eq.) at 0°C. The reaction mixture was stirred at room temperature for 16h, after completion of the reaction (monitored by TLC) the reaction mixture was quenched with sat. NH_4_Cl solution and extracted with EtOAc (2 X 50 mL). The organic layer was separated, dried over Na_2_SO_4_ and concentrated. Obtained crude residue was purified by column chromatography, eluting with 30% of EtOAc in Hexane to afford *tert*-butyl(6-bromoquinolin-3-yl) carbamate (2) (200 mg, 27.6% yield) as brown color solid.

LCMS (m/z): 92.15 % (M^+1^ = 323.1) at 5.907 RT.

**Step-2**: Synthesis of *tert*-butyl-N-(6-bromoquinolin-3-yl)-N-(*tert*-butoxycarbonyl) glycinate (3):

To a stirred solution of *tert*-butyl-N-(6-bromoquinolin-3-yl) carbamate (2) (250 mg, 0.773 mmol, 1.0 eq.) in THF (5 mL) was added 60% NaH (62 mg, 1.54 mmol, 2.0 eq.) followed by *tert*-butyl-2-bromoacetate (301 mg, 1.54 mmol, 2.0 eq.) at 0°C. The reaction mixture was stirred at room temperature for 2h, after completion of the reaction (monitored by TLC) the reaction mass was quenched with ice cold water (10 mL) and extracted with EtOAc (2 X 50 mL). The organic layer was separated, dried over Na_2_SO_4_ and concentrated. The crude residue was purified by column chromatography, eluting with 50% of EtOAc in Hexane to afford *tert*-butyl-N-(6-bromoquinolin-3-yl)-N-(*tert*-butoxycarbonyl) glycinate (3) (230 mg, 68% yield) as brown color liquid.

^1^H NMR (400 MHz, DMSO-d6): δ 8.88 (d, *J* = 2.0 Hz, 1H), 8.29 (d, *J* = 2.4 Hz, 1H), 8.15 (d, *J* = 2.4 Hz, 1H), 7.95 (d, *J* = 8.4 Hz, 1H), 7.85 (dd, *J* = 2.0, 8.8 Hz, 1H), 4.37 (s, 2H), 1.43 (s, 18H).

**Step-3**: Synthesis of *tert*-butyl-N-(*tert*-butoxycarbonyl)-N-(6-((1-cyclopropyl-3-methyl-*1H*-pyrazol-5-yl)amino)quinolin-3-yl) glycinate (4):

To a stirred solution of *tert*-butyl-N-(6-bromoquinolin-3-yl)-N-(*tert*-butoxycarbonyl)glycinate (3) (250 mg, 0.572 mmol 1 eq.) in 1,4-dioxane (7 mL) were added 1-cyclopropyl-3-methyl-*1H*-pyrazol-5-amine(117 mg, 0.858 mmol, 1.5 eq.), cesium carbonate (371 mg, 1.14 mmol, 2.0 eq.), *tris*((1E,4E)-1,5-diphenylpenta-1,4-dien-3-one)dipalladium (10.4 mg, 0.011 mmol, 0.02 eq.) and [5-(diphenylphosphanyl)-9,9-dimethyl-*9H*-xanthen-4-yl]diphenylphosphane (16.5 mg, 0.028 mmol, 0.05 eq.) at room temperature. The reaction mixture was degassed under argon for 10 min. and heated at 90 °C for 16 h. After completion of the reaction (monitored by TLC) the reaction mass was filtered on a celite bed, washed with EtOAc (50 mL). The filtrate was concentrated under reduced pressure. The crude compound was purified by column chromatography, eluting with 40% of EtOAc in Hexane to afford *tert*-butyl-N-(*tert*-butoxycarbonyl)-N-(6-((1-cyclopropyl-3-methyl-*1H*-pyrazol-5-yl) amino) quinolin-3-yl) glycinate (4) (230 mg, 81% yield) as brown color liquid.

LCMS (m/z): 94.59 % (M^+1^ = 494.3) at 9.058 RT.

**Step-4**: Synthesis of *tert*-butyl-N-(*tert*-butoxycarbonyl)-N-(6-((1-cyclopropyl-3-methyl-*1H*-pyrazol-5-yl)(methyl)amino)quinolin-3-yl)glycinate (5):

To a stirred solution of *tert*-butyl-N-(*tert*-butoxycarbonyl)-N-(6-((1-cyclopropyl-3-methyl-*1H*-pyrazol-5-yl)amino)quinolin-3-yl)glycinate (4) (230 mg, 0.466 mmol 1.0 eq.) in DMF (8 mL) was added 60% NaH (37.3 mg, 0.933 mmol, 2.0 eq.) followed by methyl iodide (0.43 mL, 0.699 mmol, 1.5 eq.) at 0°C. The reaction mixture was stirred at room temperature for 30 minutes. After completion of the reaction (monitored by TLC) the reaction mass was quenched with ice cold water (5 mL) and extracted with EtOAc (2 X 50 mL). The organic layer was separated, dried over Na_2_SO_4_ and concentrated to afford *tert*-butyl-N-(*tert*-butoxycarbonyl)-N-(6-((1-cyclopropyl-3-methyl-*1H*-pyrazol-5-yl)(methyl)amino)quinolin-3-yl) glycinate (5) (220 mg, 93% yield) as colorless liquid.

LCMS (m/z): 98.28 % (M^+1^ = 508.19) at 7.58 RT.

**Step-5**: Synthesis of (6-((1-cyclopropyl-3-methyl-*1H*-pyrazol-5-yl)methyl) amino) quinolin-3-yl) glycine (QFASG-4):

To a stirred solution of *tert*-butyl N-(*tert*-butoxycarbonyl)-N-(6-((1-cyclopropyl-3-methyl-*1H*-pyrazol-5-yl)(methyl)amino)quinolin-3-yl) glycinate (5) (230 mg, 0.453 mmol 1.0 eq.) in 1, 4-dioxane (3 mL) was added 4M. HCl in 1, 4-dioxane (3 mL) at 0°C. The reaction mixture was stirred at room temperature for 4h. After completion of the reaction (monitored by TLC) the reaction mixture was concentrated under reduced pressure to obtained crude residue. The crude compound was triturated with diethyl ether (50 mL) and *n*-pentane (50 mL) to afford (6-((1-cyclopropyl-3-methyl-*1H*-pyrazol-5-yl)(methyl)amino)quinolin-3-yl) glycine (QFASG-4) as a pale yellow solid (130 mg, 81% yield).

^1^H NMR (400 MHz, DMSO-d6): δ 13.001 (bs, 1H), 8.55 (d, *J* = 1.6 Hz, 1H), 7.88 (d, *J* = 8.2 Hz, 1H), 7.74 (s, 1H), 7.16 - 7.00 (m, 3H), 6.02 (s, 1H), 4.07 (s, 2H), 3.35 (s, 3H), 3.21 - 317 (m, 1H), 2.16 (s, 3H), 0.94 (d, *J* = 3.6 Hz, 2H), 0.75 (d, *J* = 5.2 Hz, 2H),

LCMS: 98.58 % (M^+1^ = 352.2) at 3.552 RT.

HPLC: 96.80% purity at 6.673 RT.

**QFASG-5**


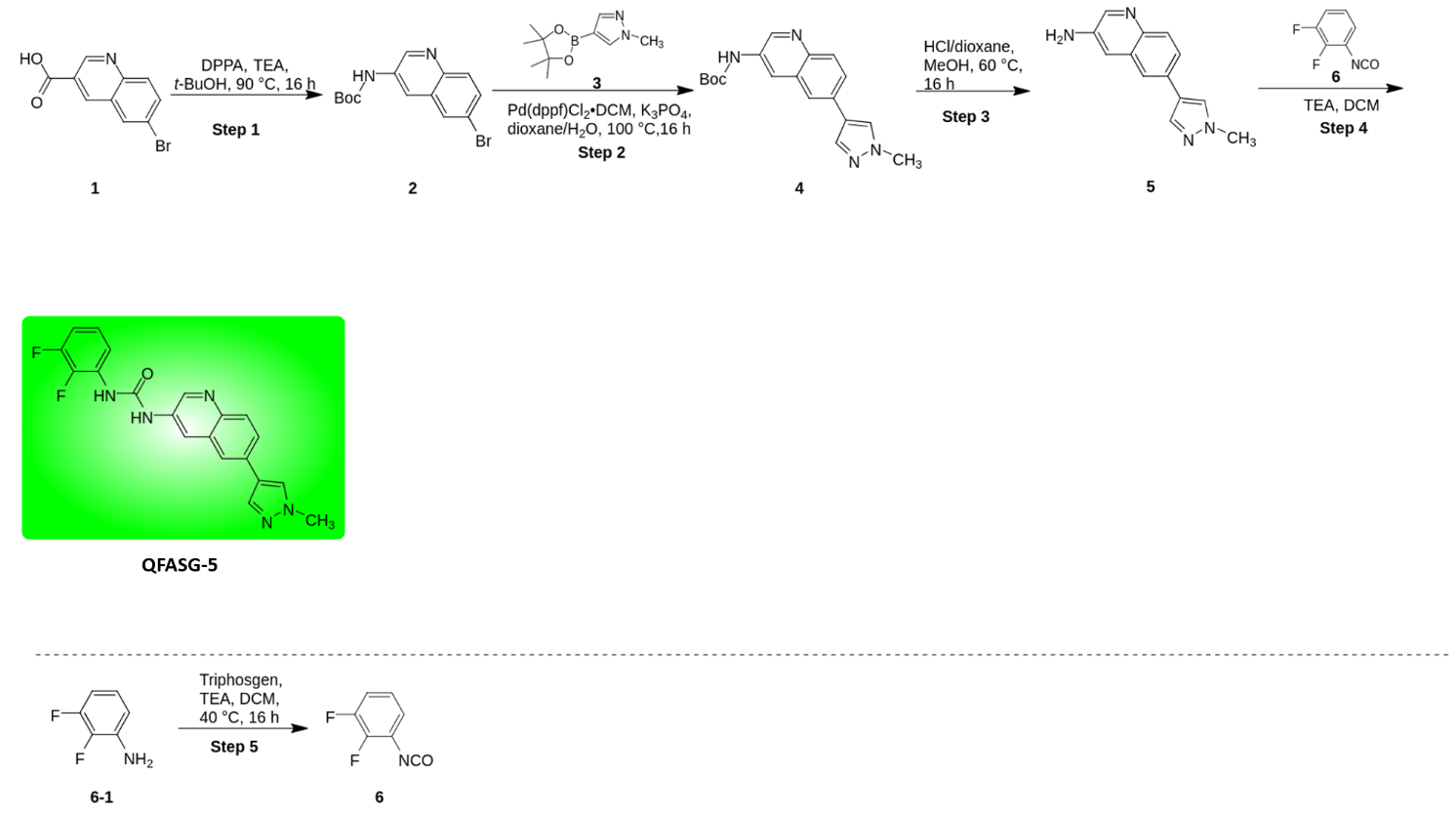


**Step 1: *tert*-butyl-N-(6-bromo-3-quinolyl)carbamate (2).** A solution of 6-bromoquinoline-3-carboxylic acid (4.5 g, 17.90 mmol) and TEA (3.62 g, 35.80 mmol, 5 mL) in *tert*-butanol (36 mL) was degassed by bubbling nitrogen for 5 min, then DPPA (9.85 g, 35.80 mmol, 8 mL) was added. The reaction mixture was stirred at 90 °C for 4 hrs. LCMS showed the starting material was consumed and new peak with desired mass was detected. The solution was partitioned between water and ethyl acetate. The aqueous layer was extracted with ethyl acetate (50 mL× 2), and the combined organics were washed sequentially with a saturated aqueous solution of sodium bicarbonate (50 mL) and brine (50 mL), dried over sodium sulfate, filtered, and concentrated in reduced pressure. The residue was further purified by flash column chromatography (silica gel, 80 g, 40% to 50% EtOAc in PE). Compound *tert*-butyl-N-(6-bromo-3-quinolyl)carbamate (2.17 g, 6.71 mmol, 37.51% yield) was obtained as yellow oil.

MS (ESI): calculated 323.0 [M (79Br) + H]^+^ , 325.0 [M (81Br) + H]^+^; measured 323.0 [M (79Br) + H]^+^ , 325.0 [M (81Br) + H]^+^

**Step 2: *tert*-butyl-N-[6-(1-methylpyrazol-4-yl)-3-quinolyl]carbamate (4).** To a solution of *tert*-butyl N-(6-bromo-3-quinolyl)carbamate (2 g, 6.19 mmol), 1-methyl-4-(4,4,5,5-tetramethyl-1,3,2-dioxaborolan-2-yl)pyrazole (1.55 g, 7.43 mmol) in 1,4-dioxane (30 mL) and water (2 mL) was added Pd(dppf)Cl_2_·CH_2_Cl_2_ (505.37 mg, 618.84 μmol), K_3_PO_4_ (3.28 g, 15.47 mmol), the mixture was degassed and bubbling with N_2_ (2 min), then it was stirred at 100 °C for 16 hrs. LCMS showed the starting materials were consumed and new peak with desired mass was detected. The solution was poured into water (50 mL), and the mixture was extracted with ethyl acetate (50 mL × 2), the combined organic layer was washed with brine, dried over Na_2_SO_4_, filtered and concentrated under reduced pressure to give a residue. The residue was further purified by flash column chromatography (silica gel, 24 g, 70% to 95% EtOAc in PE). Compound *tert*-butyl-N-[6-(1-methylpyrazol-4-yl)-3-quinolyl] carbamate (1.3 g, 4.00 mmol, 64.6% yield) was obtained as a white solid.

MS (ESI): calculated 325.2 [(M + H)^+^]; measured 325.2 [(M + H)^+^]

**Step 3: 6-(1-methylpyrazol-4-yl)quinolin-3-amine (5).** To a solution of *tert*-butyl-N-[6-(1-methylpyrazol-4-yl)-3-quinolyl]carbamate (0.5 g, 1.54 mmol) in MeOH (10 mL) was added HCl/1,4-dioxane (4 M, 4 mL), the mixture was stirred at 60 °C for 16 hrs. Then the solution was concentrated under reduced pressure to give 6-(1-methylpyrazol-4-yl)quinolin-3-amine (0.4 g, 1.53 mmol, 99.53% yield, HCl) as a white solid, which was used for next step directly.

**Step 4: 1-(2,3-difluorophenyl)-3-[6-(1-methylpyrazol-4-yl)-3-quinolyl]urea (QFASG-5).** To a solution of 2,3-difluoroaniline (3 g, 23.24 mmol, 2.36 mL) in toluene (30 mL) was added TEA (5.88 g, 58.09 mmol, 8 mL), *bis*(trichloromethyl) carbonate (2.34 g, 7.90 mmol), the mixture was stirred at 40 °C for 16 hrs. The reaction mixture was filtered and the filtrate was collected to give 1,2-difluoro-3-isocyanato-benzene (3 g, crude product) as colorless liquid which was used for next step directly without purification. To a solution of 6-(1-methylpyrazol-4-yl)quinolin-3-amine (0.4 g, 1.53 mmol, 1 eq, HCl) in DCM (20 mL) was added TEA (466 mg, 4.60 mmol), then 1,2-difluoro-3-isocyanato-benzene (357 mg, 2.30 mmol) was added to the mixture, the mixture was stirred at 25°C for 1 hr. LCMS showed the starting material was consumed and new peak with desired mass was detected. The solution was poured into water (50 mL), and the mixture was extracted with DCM (50 mL × 3), the combined organic layer was washed with brine, dried over Na_2_SO_4_, filtered and concentrated under reduced pressure to give a residue. The residue was purified by prep-HPLC (column: Waters Xbridge 150*25mm*5µm; mobile phase: [water (NH_4_HCO_3_) - ACN]; gradient: 32%-62% B over 8 min) and lyophilized. Compound 1-(2,3-difluorophenyl)-3-[6-(1-methylpyrazol-4-yl)-3-quinolyl]urea (22.04 mg, 58.10 µmol, 3.79% yield) was obtained as a white solid.

MS (ESI): calculated 380.1 [(M + H)^+^]; measured 380.1 [(M + H)^+^]

^1^H NMR (400 MHz, DMSO-d6) δ ppm: 9.54 (s, 1 H), 8.96 (s, 1 H), 8.72 (d, *J* = 2.4 Hz, 1H), 8.51 (d, *J* = 2.0 Hz, 1H), 8.30 (s, 1 H), 8.09 (d, *J* = 2.0 Hz, 1H), 8.04 (s, 1 H), 7.99 (s, 1 H), 7.93-7.90 (m, 1 H), 7.90-7.86 (m, 1 H), 7.20-7.18 (m, 1 H), 7.10-7.09 (m, 1 H) , 3.91 (s, 3 H)

**QFASG-6**

**
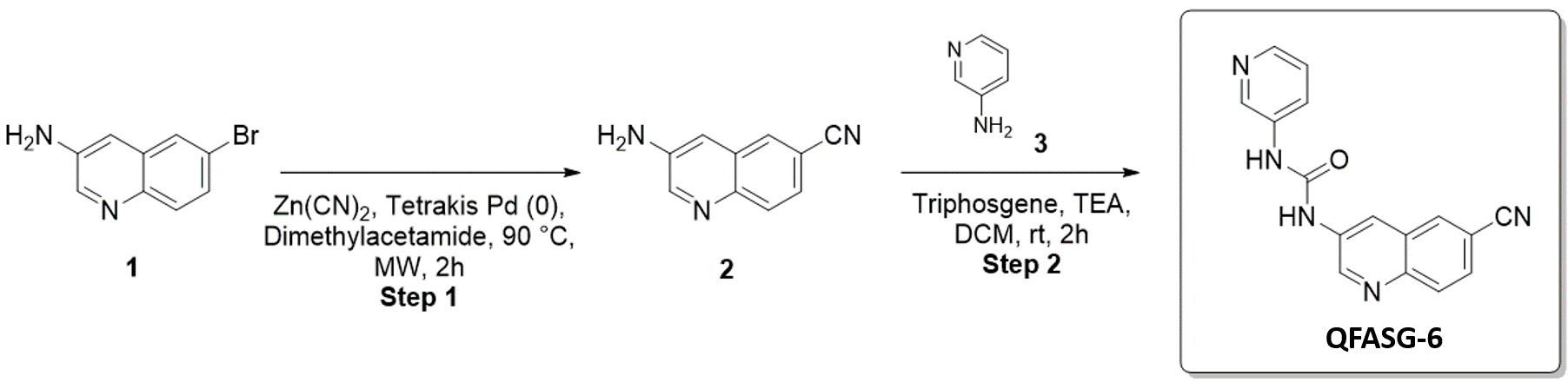
**

**Step-1**: Synthesis of 3-aminoquinoline-6-carbonitrile (2):

To a stirred solution of 6-bromoquinolin-3-amine (1) (100 mg, 0.448 mmol, 1.0 eq.) in dimethylacetamide (2 mL) was added zinc cyanide (304 mg, 2.242 mmol, 5.0 eq.) and palladium-*tetrakis*(triphenylphosphine) (155 mg, 0.134 mmol, 0.3 eq.) at room temperature in a microwave vial. The reaction mixture was heated at 90°C in a microwave for 2h. After completion of the reaction (monitored by TLC) the reaction mixture was diluted with water (10 mL) and EtOAc (50 mL) then filtered on a celite bed washed with EtOAc (20 mL). The organic layer was separated, dried over Na_2_SO_4_ and concentrated. The crude compound was purified by column chromatography, eluting with 40% of EtOAc in Hexane to afford 3-aminoquinoline-6-carbonitrile (2) as pale yellow liquid (70 mg, 84% pure).

LCMS: 84.51 % (M^+1^ = 170.10) at 4.44 RT.

**Step-2**: Synthesis of 1-(6-cyanoquinolin-3-yl)-3-(pyridin-3-yl) urea (QFASG-6):

To a stirred solution of 3-aminoquinoline-6-carbonitrile (2) (120 mg, 0.71 mmol, 1.0 eq.) in dichloromethane (4 mL) was added triphosgene (420 mg, 1.42 mmol, 2.0 eq.) and triethylamine (0.18 mL, 1.42 mmol, 2.0 eq.) followed by pyridin-3-amine (80 mg, 0.85 mmol, 1.2 eq.) at 0°C. The reaction mixture was stirred at room temperature for 2h. After completion of the reaction (monitored by TLC) the reaction mixture was diluted with water (10 mL) and extracted with DCM (2 X 50 mL). The organic layer was separated, dried over Na_2_SO_4_ and concentrated to afford the crude product. Obtained crude product was purified by prep-HPLC using 0.1% formic acid + ACN (0-30%, gradient elution, Column-Luna C^18^, 250 x 50 mm, 10 µm) to get 1-(6-cyanoquinolin-3-yl)-3-(pyridin-3-yl)urea (QFASG-6) as off-white solid (10 mg, yield: 18.9%).

^1^H NMR (400 MHz, DMSO-d6): δ 9.77 (s, 1H), 9.43 (s, 1H), 9.01 (s, 1H), 8.67 (d, *J* = 2 Hz, 2H), 8.62 (s, 1H), 8.24 (d, *J* = 4.4 Hz, 1H), 8.09 (d, *J* = 8.4 Hz, 1H), 7.99 (d, *J* = 8.0 Hz, 1H), 7.88 (d, *J* = 8.4 Hz, 1H), 7.37 – 7.34 (m, 1H),

LCMS: 98.88 % (M^+1^ = 352.2) at 3.524 RT.

HPLC: 98.95% purity at 6.404 RT.

**Supplementary Data 2.** Setup for reproducing known protein ligands binding poses.

Number of probes for DFT-driven position optimization – 4

Energy threshold for selection - 0.5

Number of entries for rigid docking – 1000

Selection via calculated binding energy

Shape similarity - Off

IFP clustering – Off

Number of structures for next iteration - 15

Maximum selected conformations per structure – 1

Probe crucial filter:

interaction_limit: 3.5

h_interaction_limit: 2.5

Request - individual

Crucial filter:

interaction_limit: 3.5

h_interaction_limit: 2.5

Request - individual

Alignment - rms limit – 2.0

**Supplementary Data 3.** CAMKK2 generation setup.

Number of probes for DFT-driven position optimization - 2

Energy threshold for selection - 0.5

Iterations number - 6

Number of entries for rigid docking - 500

Selection via calculated binding energy

Shape similarity - Off

IFP clustering - On

Number of top-scored structures selected for clustering - 250

Number of structures for next iteration - 10

Maximum selected conformations per structure - 1

Probe crucial filter:

Distance between heavy atoms - 3.5

Distance to hydrogen - 2.5

Request - '270_N_acceptor'

Crucial filter:

Distance between heavy atoms - 3.5

Distance to hydrogen - 2.5

Request - '270_N_acceptor'

Alignment - rms limit - 1.5

**Supplementary Data 4.** ATM generation setup.

Number of probes for DFT-driven position optimization - 3

Energy threshold for selection - 0.5

Iterations number - 4

Number of entries for rigid docking - 200

Selection via calculated binding energy

Shape similarity - Off

IFP clustering - On

Number of top-scored structures selected for clustering - 50

Number of structures for next iteration - 4

Maximum selected conformations per structure - 2

Probe crucial filter:

Distance between heavy atoms - 3.5

Distance to hydrogen - 2.5

Request - '2770_N_acceptor'

Crucial filter:

Distance between heavy atoms - 3.5

Distance to hydrogen - 2.5

Request - '2770_N_acceptor'

Alignment - rms limit - 1.5

# Supplementary Figures and Tables

## Supplementary Figures

#
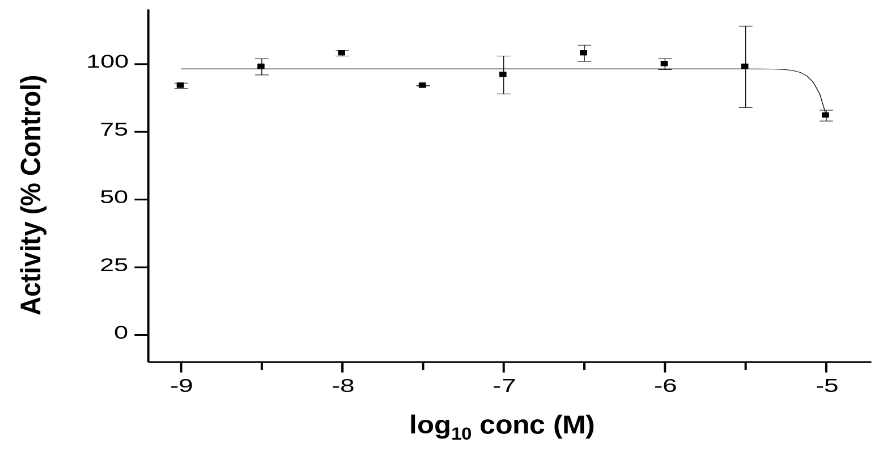


# Supplementary Figure 1. Representative dose–response curve for QFASG-1 in CAMKK2 activity assay.

#
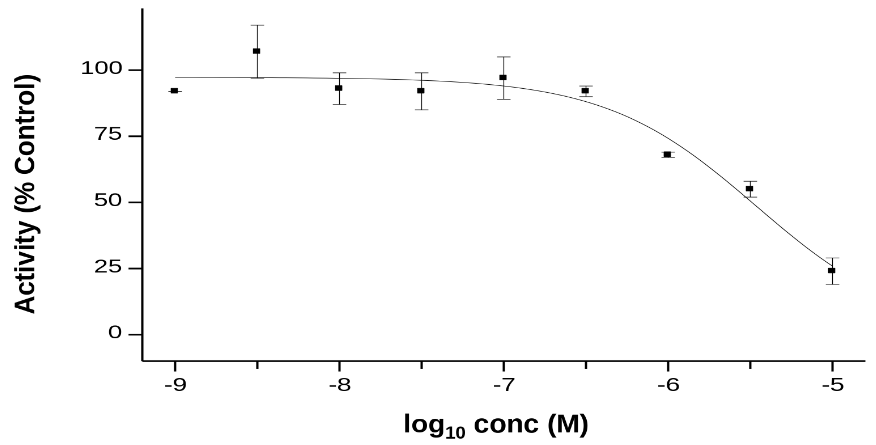


# Supplementary Figure 2. Representative dose–response curve for QFASG-2 in CAMKK2 activity assay.

#
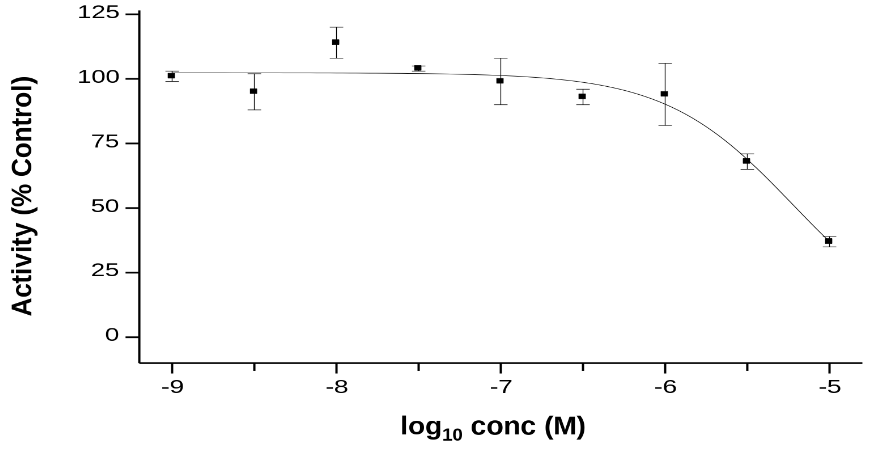


# Supplementary Figure 3. Representative dose–response curve for QFASG-3 in CAMKK2 activity assay.

#
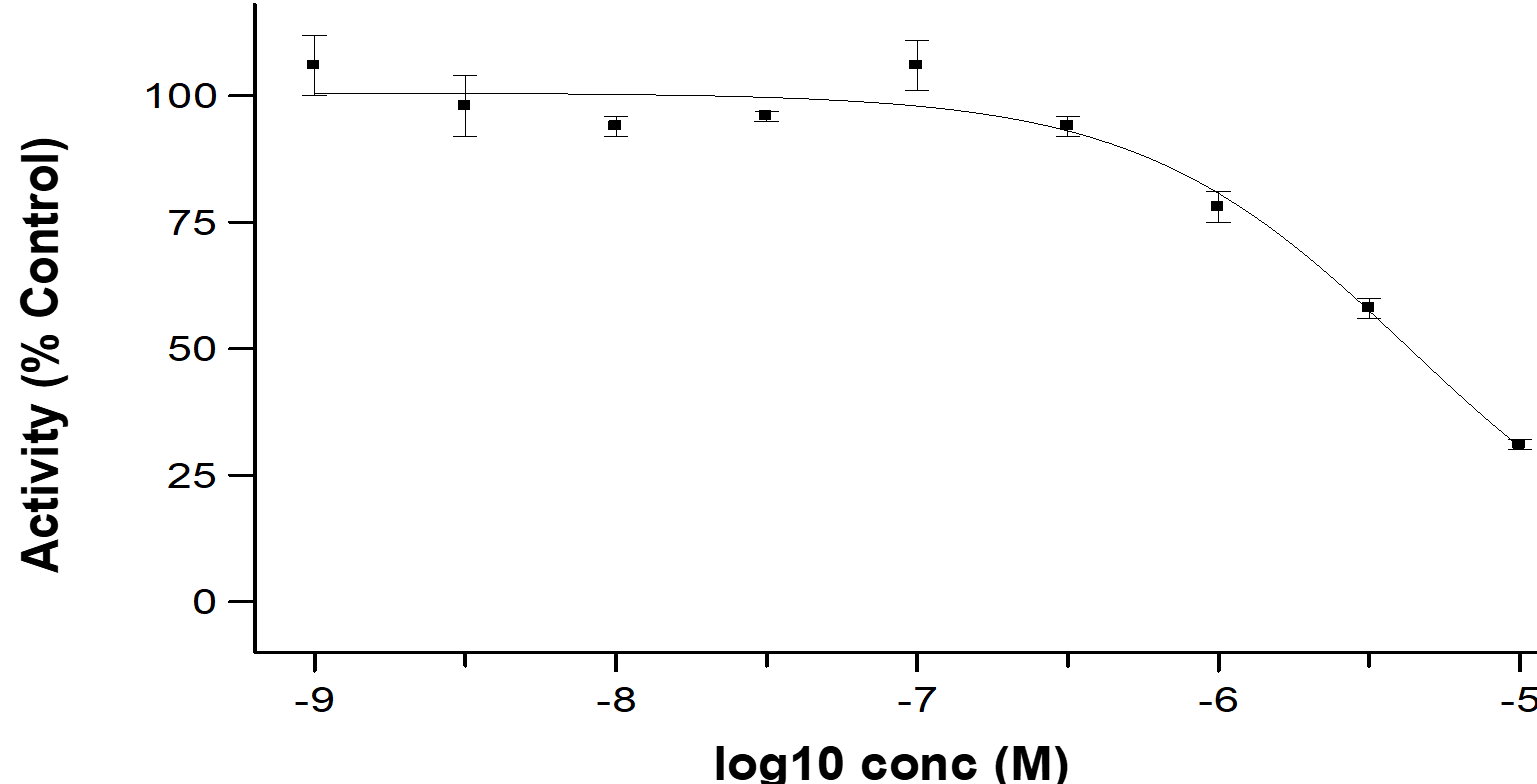


# Supplementary Figure 4. Representative dose–response curve for QFASG-6 in ATM activity assay.

#
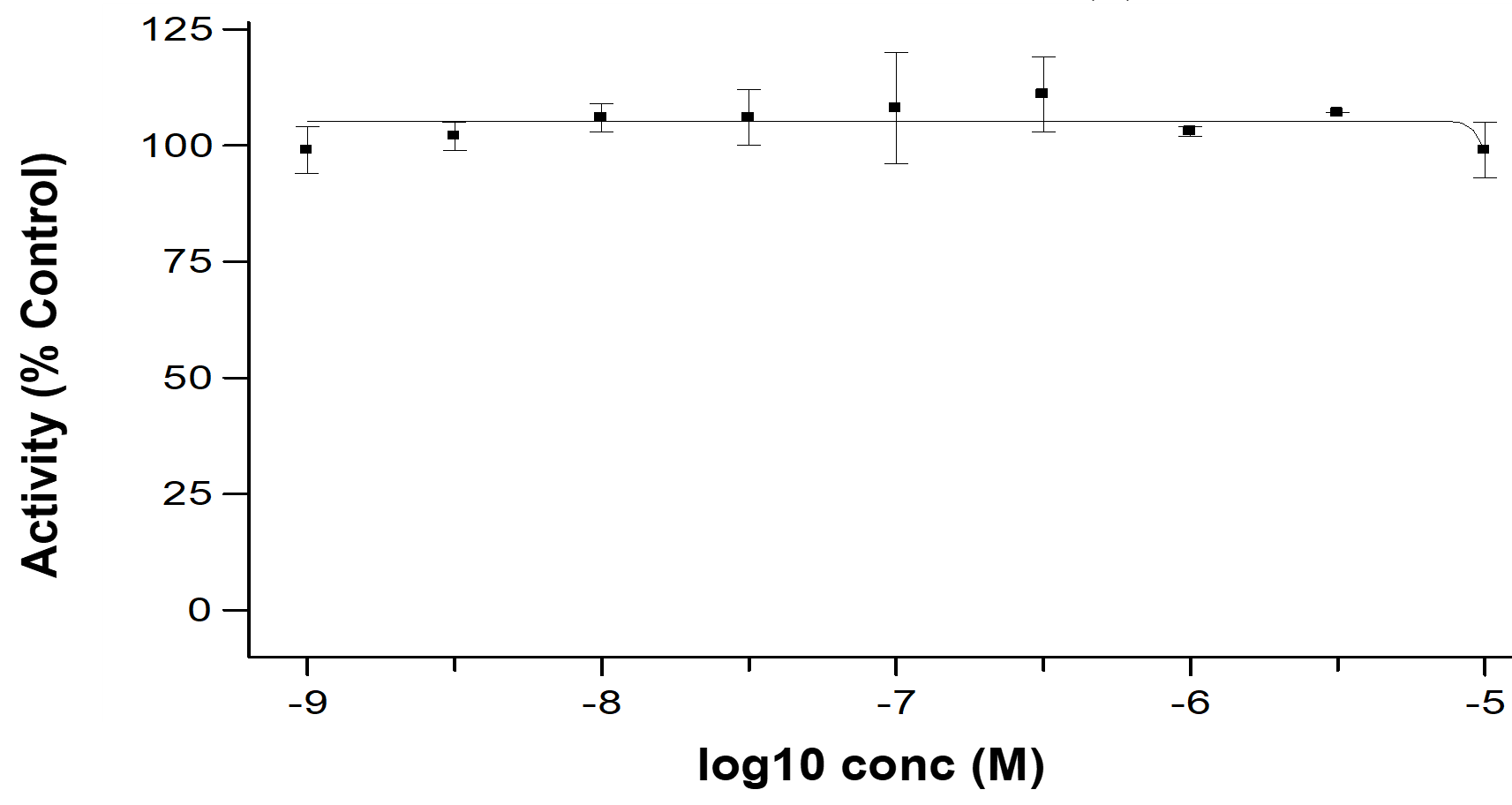


# Supplementary Figure 5. Representative dose–response curve for QFASG-6 in ATR activity assay.

#
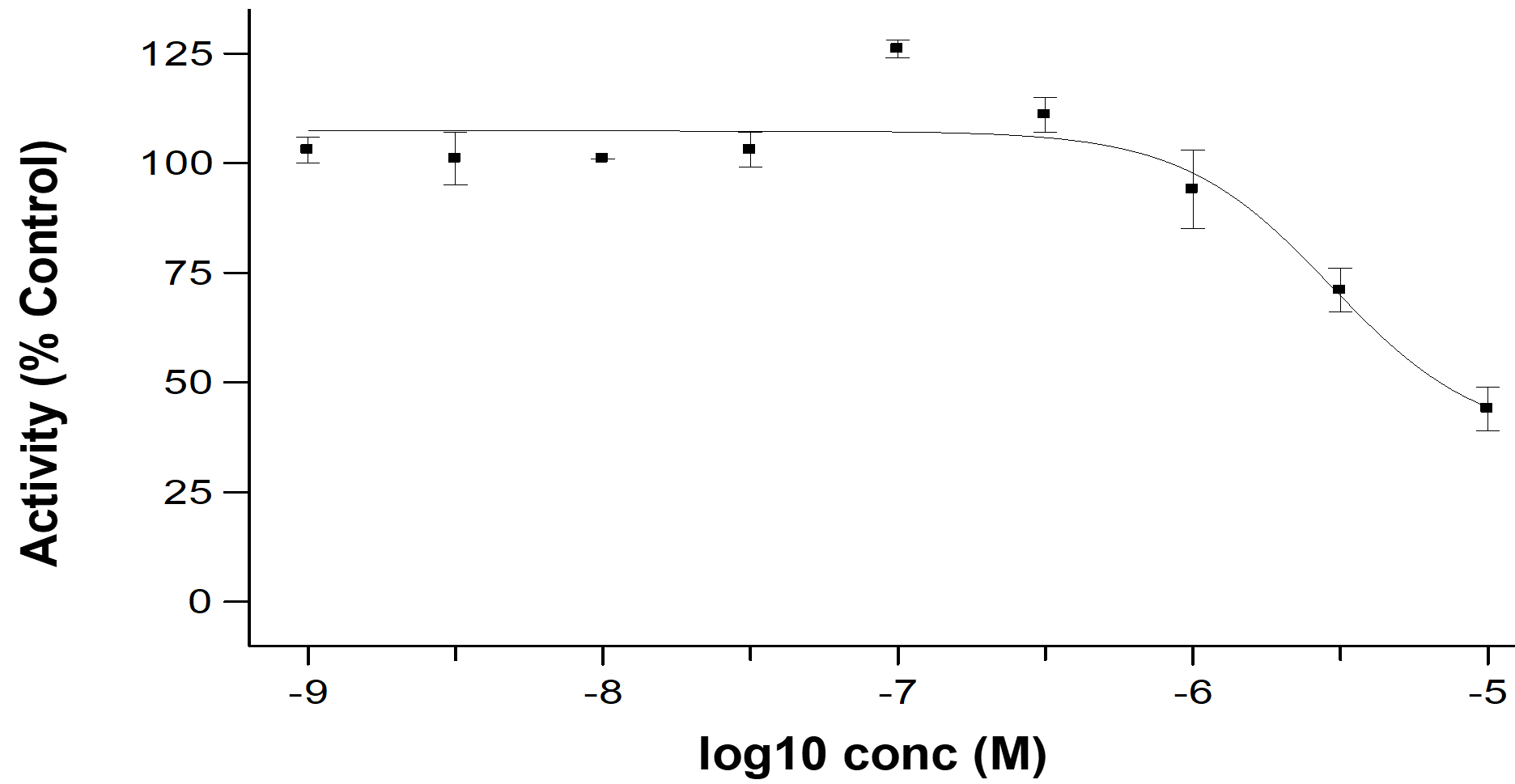


# Supplementary Figure 6. Representative dose–response curve for QFASG-6 in DNA-PK activity assay.


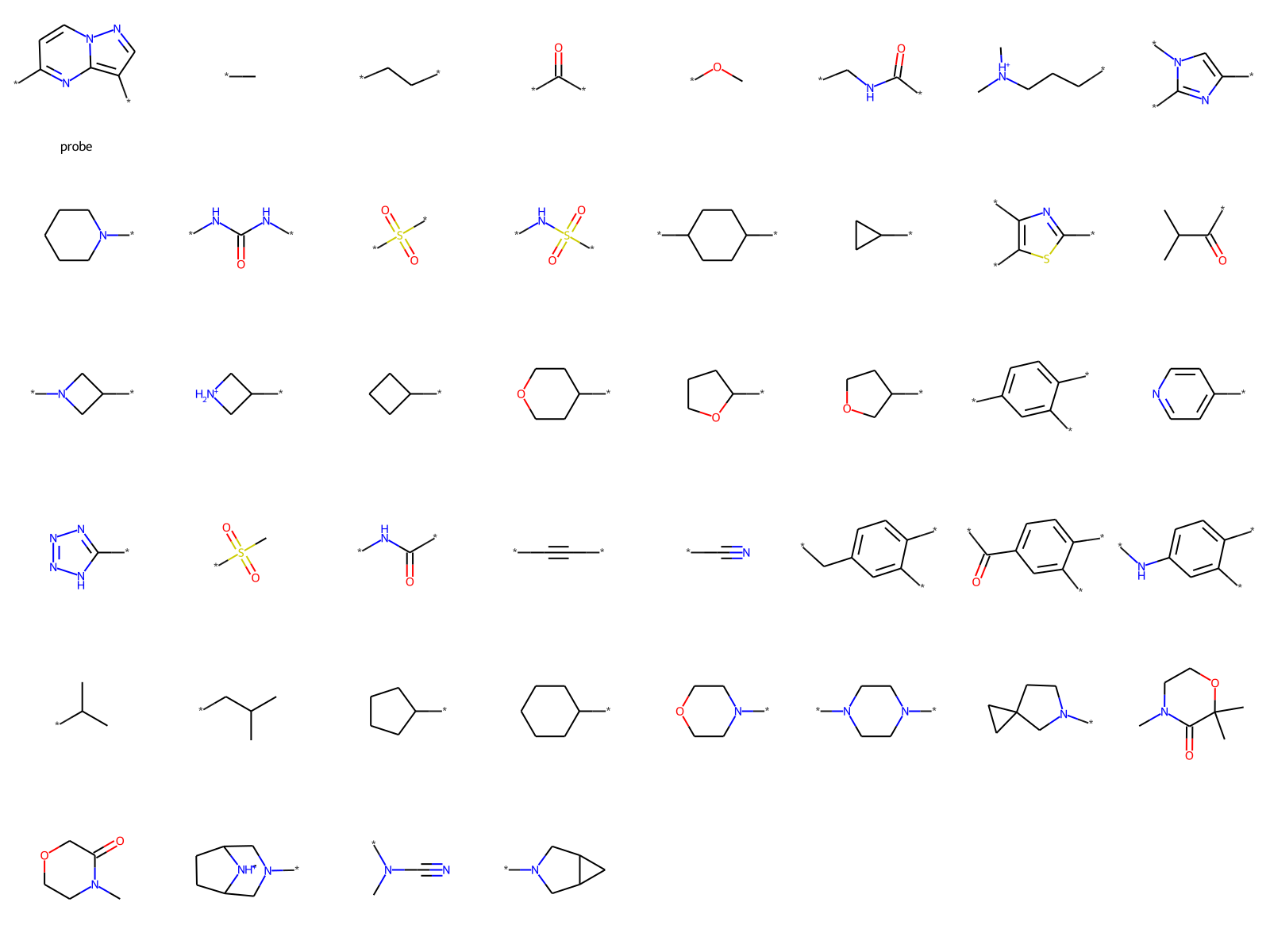


**Supplementary Figure 7.** CAMKK2 probe and linkers.


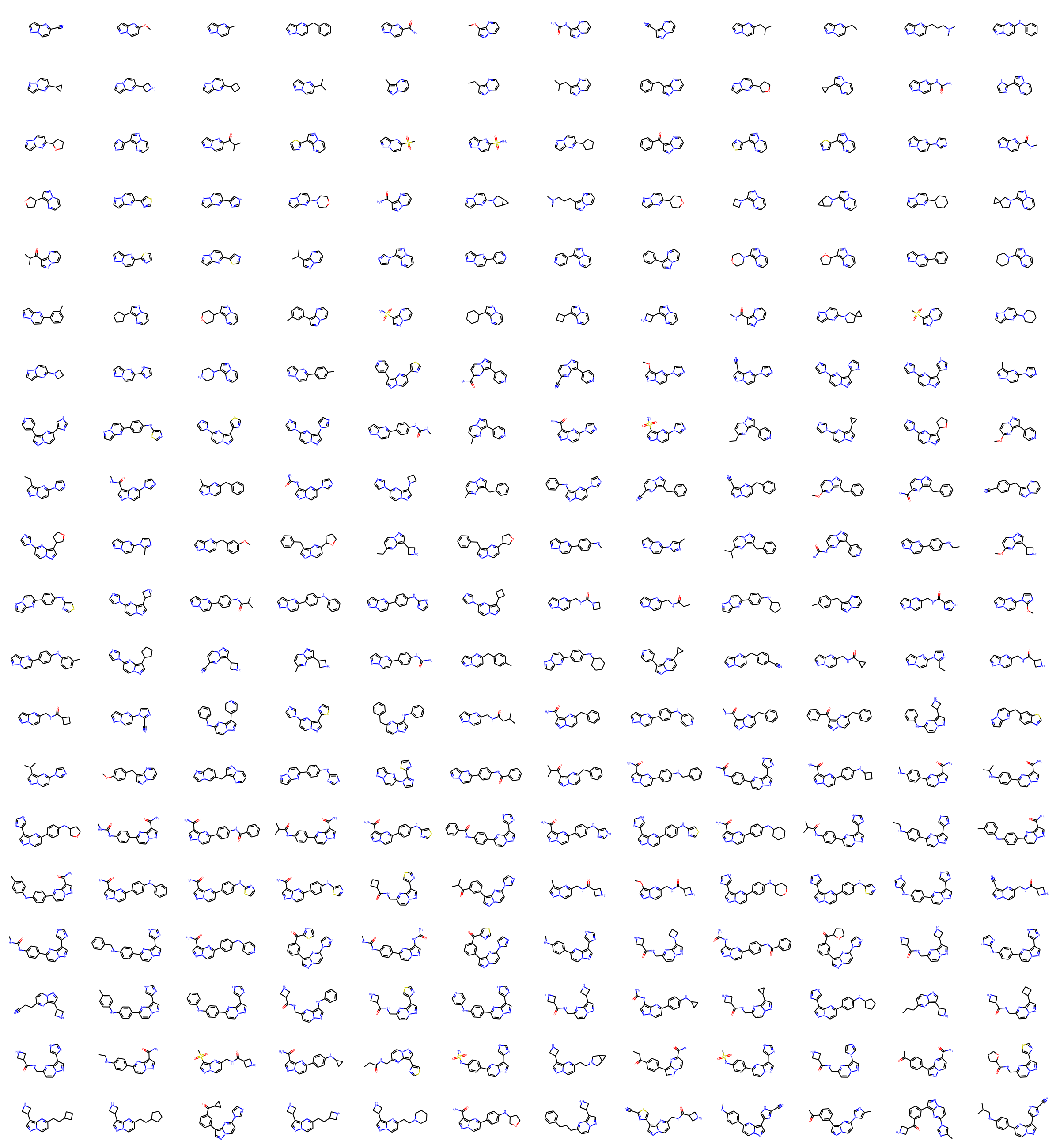


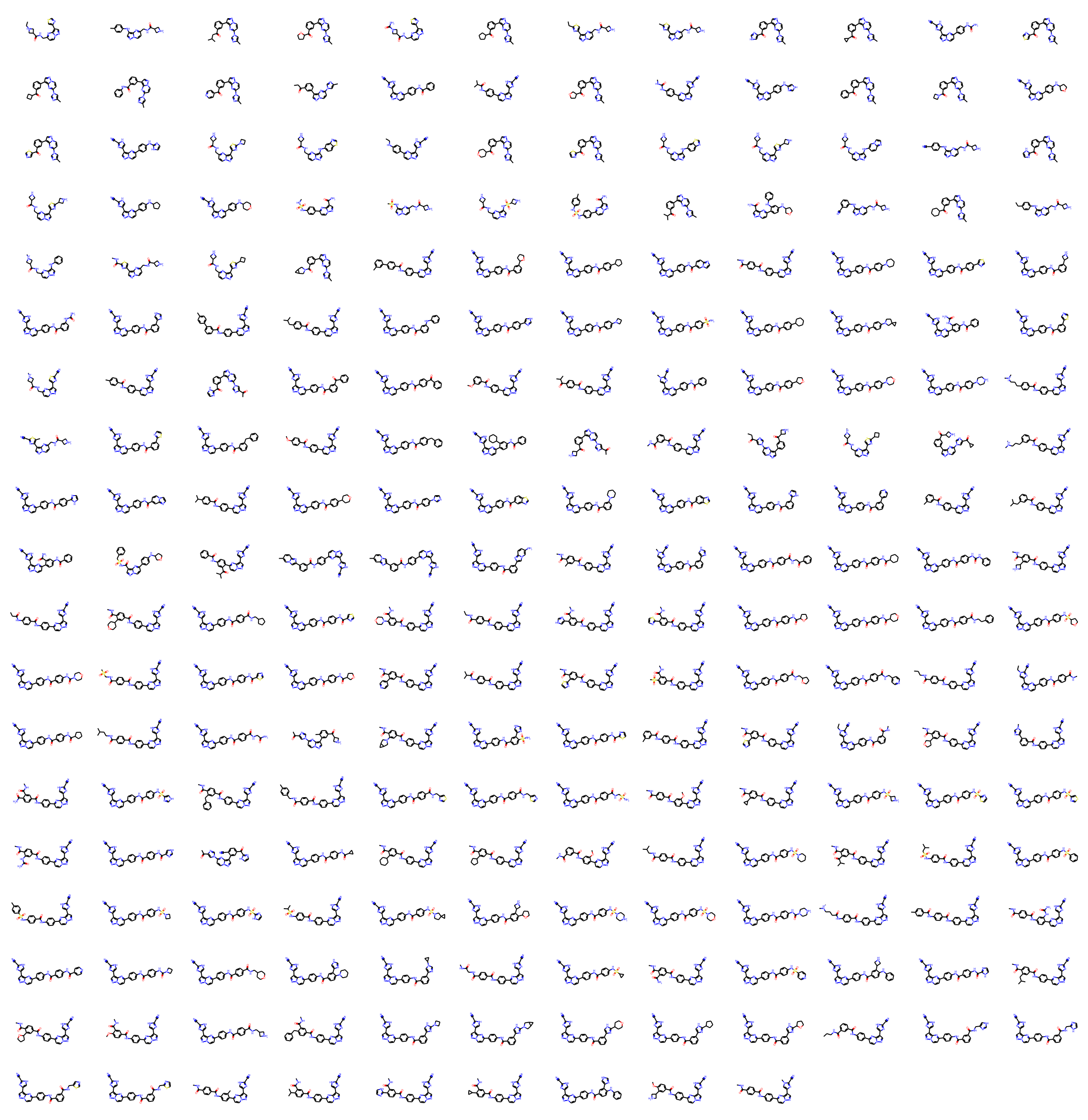


**Supplementary Figure 8.** CAMKK2 inhibitors generation results.


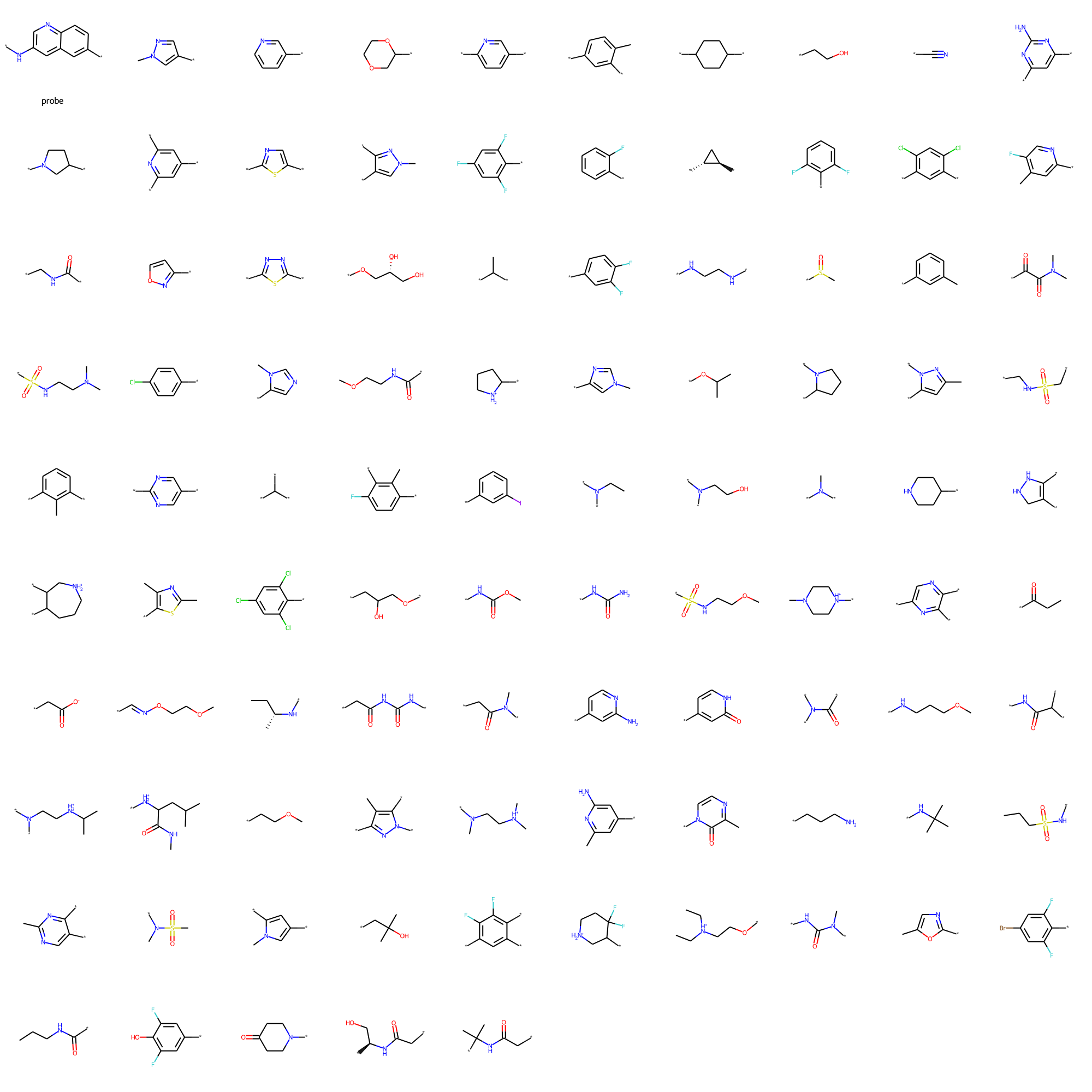


**Supplementary Figure 9.** ATM probe and linkers.


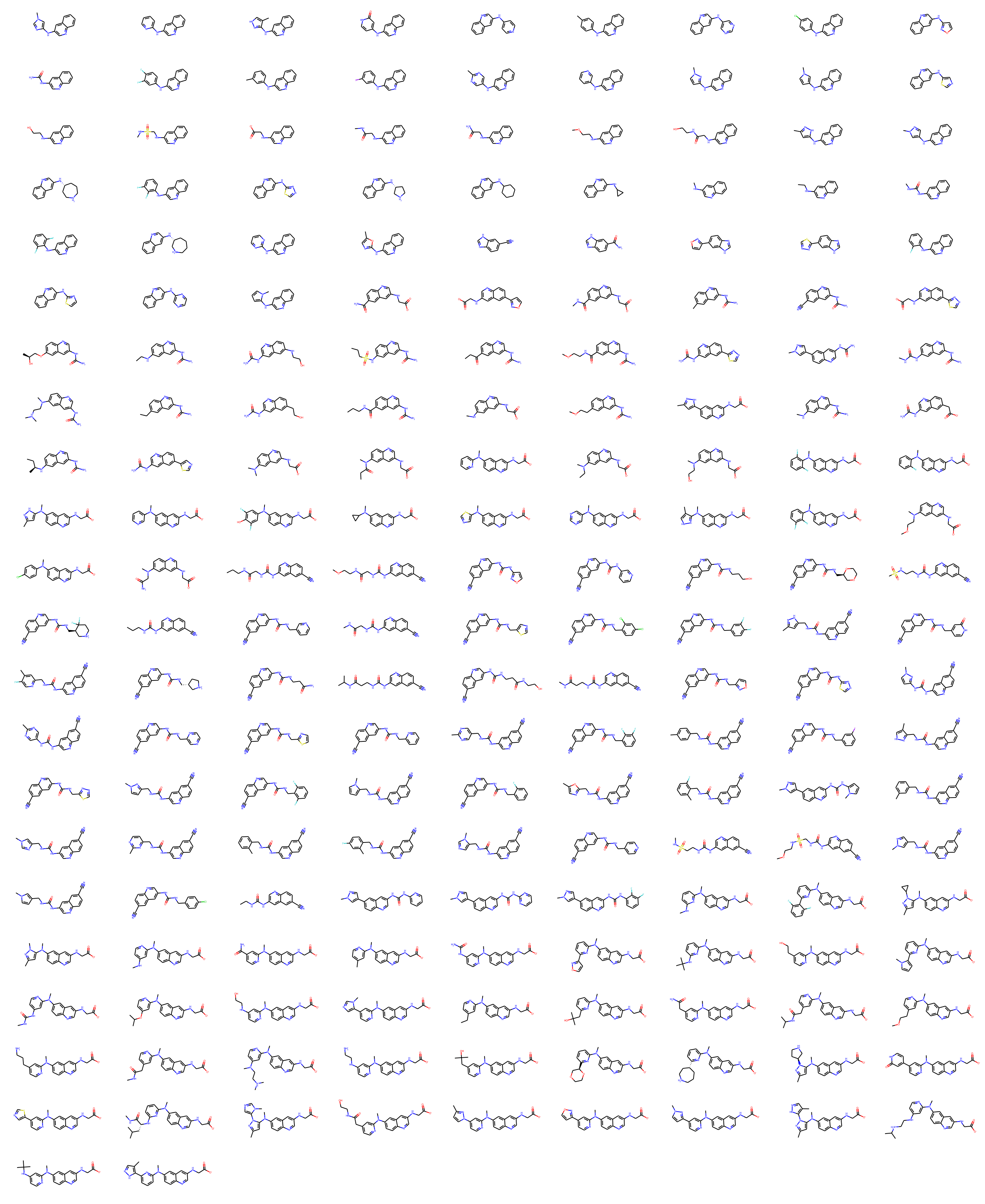


**Supplementary Figure 10.** ATM inhibitors generation results.

## Supplementary Tables

### Supplementary Table 1. Comparison with known methods

| **Feature** | **QFASG (2024)** | **ACFIS (2016)** | **AutoGrow4 (2020)** | **LigBuilder V3 (2020)** |
| --- | --- | --- | --- | --- |
| *De novo* design | + | - | + | + |
| Binding Site Mapping | + | - | + | + |
| Comprehensive linking rules | + | - | + | - |
| Interaction-based diversity | + | - | - | - |
| Drug-likeness evaluation | + | - | + | + |
| Metabolic stability optimization | + | - | - | - |
| Synthetic accessibility estimation | + | - | - | + |
| Novelty of structures constructed | + | - | - | - |
| Don’t need external software | + | - | - | - |

# Supplementary Table 2. Results of binding mode reproduction for known ligands

| PDB ID | Best RMSD | Number of heavy atoms | Number of rotatable bonds |
| --- | --- | --- | --- |
| 3S73 | 0.36 | 13 | 1 |
| 1AZM | 0.42 | 13 | 2 |
| 3S71 | 0.64 | 13 | 1 |
| 4GKI | 0.91 | 25 | 3 |
| 2XN5 | 0.99 | 21 | 5 |
| 5J3L | 1.08 | 15 | 4 |
| 5J64 | 1.17 | 21 | 2 |
| 4MYD | 2.22 | 17 | 5 |
| 1CBX | 2.29 | 15 | 5 |
| 4JX9 | 2.71 | 17 | 2 |
| 5MG2 | 2.78 | 32 | 4 |
| 1G46 | 2.83 | 22 | 4 |
| 1X38 | 3.32 | 20 | 2 |
| 3OAF | 4.07 | 22 | 3 |
| 3HKU | 4.29 | 22 | 3 |
| 4EFS | 8.51 | 29 | 8 |
| 2OGY | 11.5 | 33 | 9 |
